# Genome-Wide CRISPR-Cas9 Screen Identifies SMCHD1 as a Restriction Factor for Herpesviruses

**DOI:** 10.1128/mbio.00549-23

**Published:** 2023-04-03

**Authors:** Xuezhang Tian, Yaru Zhou, Shaowei Wang, Ming Gao, Yanlin Xia, Yangyang Li, Yunhong Zhong, Wenhao Xu, Lei Bai, Bishi Fu, Yu Zhou, Hye-Ra Lee, Hongyu Deng, Ke Lan, Pinghui Feng, Junjie Zhang

**Affiliations:** a State Key Laboratory Breeding Base of Basic Science of Stomatology & Key Laboratory of Oral Biomedicine Ministry of Education, School & Hospital of Stomatology, State Key Laboratory of Virology, Medical Research Institute, Wuhan University, Wuhan, China; b Frontier Science Center for Immunology and Metabolism, Wuhan University, Wuhan, China; c Department of Pulmonary and Critical Care Medicine, Zhongnan Hospital of Wuhan University, Wuhan, China; d Wuhan Research Center for Infectious Diseases and Cancer, Chinese Academy of Medical Sciences, Wuhan, China; e State Key Laboratory of Virology, School of Life Sciences, Wuhan University, Wuhan, China; f Department of Biotechnology and Bioinformatics, College of Science and Technology, Korea University, Sejong, South Korea; g Department of Lab Medicine, College of Medicine, Korea University, Seoul, South Korea; h CAS Key Laboratory of Infection and Immunity, CAS Center for Excellence in Biomacromolecules, Institute of Biophysics, Chinese Academy of Sciences, Beijing, China; i University of Chinese Academy of Sciences, Beijing, China; j Section of Infection and Immunity, Herman Ostrow School of Dentistry, Norris Comprehensive Cancer Center, University of Southern California, Los Angeles, California, USA; University of North Carolina at Chapel Hill

**Keywords:** DNA replication, KSHV, Ori-Lyt, SMCHD1, herpesvirus, restriction factor

## Abstract

Intrinsic immunity is the frontline of host defense against invading pathogens. To combat viral infection, mammalian hosts deploy cell-intrinsic effectors to block viral replication prior to the onset of innate and adaptive immunity. In this study, SMCHD1 is identified as a pivotal cellular factor that restricts Kaposi’s sarcoma-associated herpesvirus (KSHV) lytic reactivation through a genome-wide CRISPR-Cas9 knockout screen. Genome-wide chromatin profiling revealed that SMCHD1 associates with the KSHV genome, most prominently the origin of lytic DNA replication (ORI-Lyt). SMCHD1 mutants defective in DNA binding could not bind ORI-Lyt and failed to restrict KSHV lytic replication. Moreover, SMCHD1 functioned as a pan-herpesvirus restriction factor that potently suppressed a wide range of herpesviruses, including alpha, beta, and gamma subfamilies. SMCHD1 deficiency facilitated the replication of a murine herpesvirus *in vivo*. These findings uncovered SMCHD1 as a restriction factor against herpesviruses, and this could be harnessed for the development of antiviral therapies to limit viral infection.

## INTRODUCTION

Herpesviruses are highly prevalent in the human population, and herpesvirus infection causes significant morbidity and mortality, ranging from blisters to lethal encephalitis and cancer, especially in immunocompromised individuals. *Herpesviridae* is divided into three subfamilies, *Alphaherpesvirinae*, *Betaherpesvirinae*, and *Gammaherpesvirinae*; despite differences in tissue tropism and genetic organization, herpesviruses share common life cycle and replication strategies. Herpesviruses are able to establish lifelong latency in infected hosts, and the switch from latency to lytic replication is one of the most notable features of the herpesvirus life cycle, as it is accompanied by viral DNA replication and infectious virion production (1). Although viral factors regulating lytic reactivation have been extensively investigated, our understanding of host factors that modulate herpesvirus lytic replication remains very limited.

To control herpesvirus infection, mammalian hosts deploy a large number of protein-based antiviral effectors. These effectors can be roughly divided into two groups: interferon (IFN)-induced effectors and cell-intrinsic effectors. Many antiviral effectors are encoded by interferon-induced genes (ISGs) and are induced by interferon stimulation. These interferon-induced effectors elicit antiviral responses against a wide range of viruses, including herpesviruses (2–4). For example, MxB is an interferon-induced restriction factor that blocks multiple steps of the herpesvirus infection cycle (5, 6). Interferon-induced tetratricopeptide repeat-containing proteins (IFITs) are among the most highly interferon-induced proteins, and they potently restrict Kaposi’s sarcoma-associated herpesvirus (KSHV) lytic replication (7). However, our understanding of cell-intrinsic antiviral effectors that modulate herpesvirus replication is still rudimentary.

In this study, we identified structural maintenance of chromosomes flexible hinge domain-containing protein 1 (SMCHD1) as a restriction factor for KSHV lytic reactivation via a genome-wide CRISPR-Cas9 knockout screen. SMCHD1 is a well-characterized epigenetic regulator that plays a critical role in development, but its function in herpesvirus infection remains unknown (8). We found that SMCHD1 associates with the origin of lytic DNA replication (ORI-Lyt), and the hinge domain of SMCHD1 is required for targeting ORI-Lyt and restricting KSHV lytic replication. Notably, SMCHD1 is a pan-herpesvirus restriction factor that suppresses the replication of a wide range of herpesviruses, including the alpha, beta, and gamma subfamilies. SMCHD1 deficiency facilitates the replication of a murine herpesvirus *in vivo*. These findings uncovered SMCHD1 as a herpesvirus restriction factor, which can be harnessed to develop new therapeutics for the treatment of herpesvirus infection and the related diseases.

## RESULTS

### Identification of SMCHD1 as a restriction factor for KSHV lytic reactivation.

**FIG 1:**
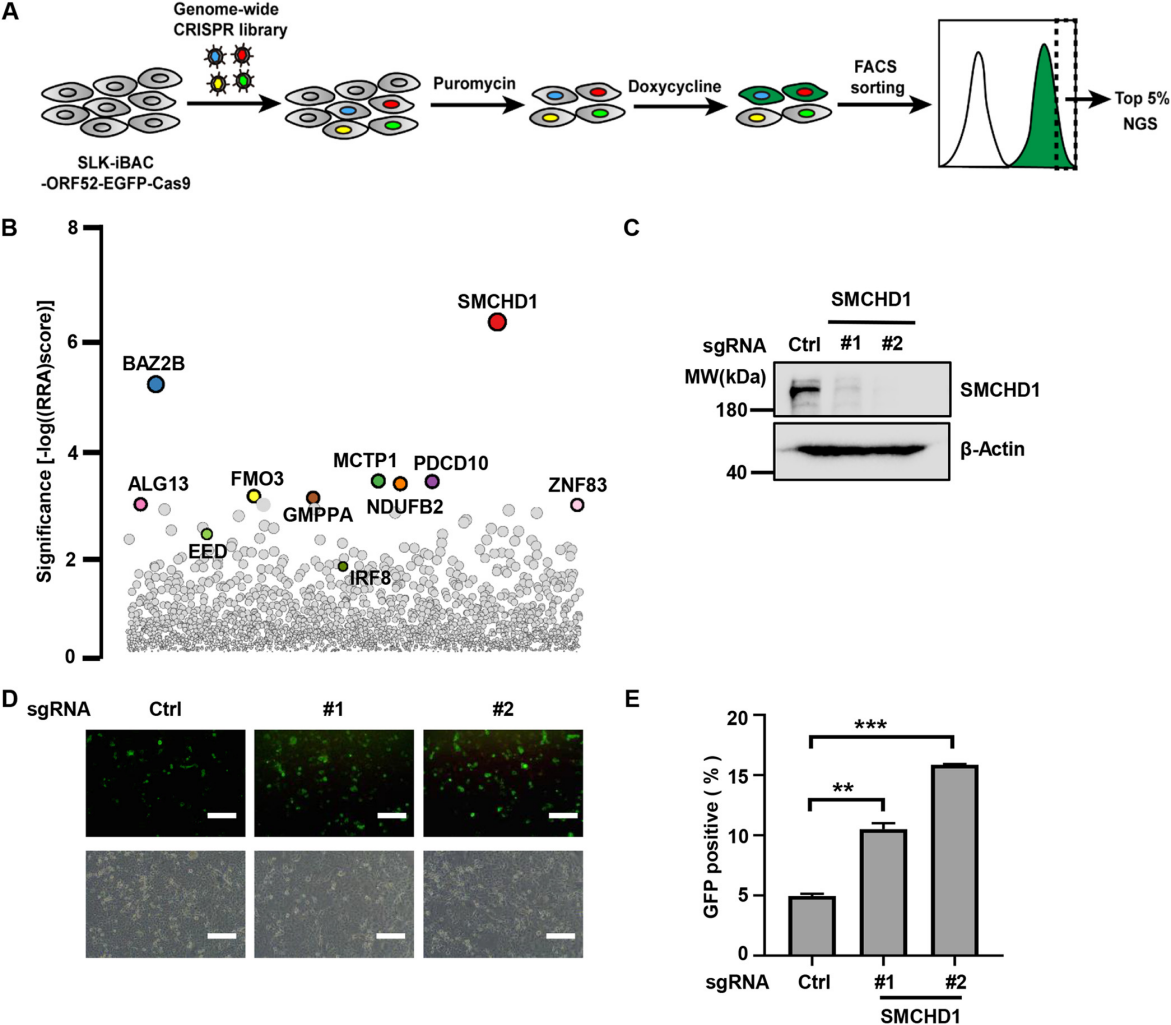
Genome-wide CRISPR knockout screen identified SMCHD1 as a restriction factor for KSHV reactivation. (A) CRISPR screen workflow. Cas9^+^ SLK.iBAC-ORF52-EGFP cells were transduced with the GeCKO v2 sgRNA library, followed by selection with puromycin. The cell pools were induced with doxycycline to initiate reactivation, followed by sorting of the cells with top 5% EGFP intensity for next-generation sequencing. Lentivirus-integrated sgRNA abundances from the sorted cells versus input were quantitated. (B) Bubble plots indicating the genes that were significantly enriched in the screening. Highlighted genes are the most potential restriction factors that were tested in this study. (C) SLK.iBAC-ORF52-GFP cells were transduced with sgRNA targeting SMCHD1 to generate knockout cells. Whole-cell lysates (WCLs) were analyzed by immunoblotting. (D) Control or *SMCHD1*^−/−^ SLK.iBAC-ORF52-GFP cells were induced with Dox, and GFP expression was imaged 24 h postinduction. Scale bars, 100 μm. (E) SLK.iBAC-ORF52-GFP stable cells were treated as described for panel D, and the percentages of GFP-positive cells were quantified through flow cytometry analysis.

To identify host factors that regulate herpesvirus lytic reactivation, we performed a genome-wide CRISPR-Cas9 knockout screen in SLK-iBAC-ORF52-EGFP cells. SLK-iBAC-ORF52-EGFP cells (here referred to as reporter cells) are derived from the parental SLK cells and are latently infected with KSHV that has been engineered to initiate lytic reactivation from a doxycycline-inducible promoter (9). Furthermore, open reading frame 52 (ORF52), encoded by a true late gene, has been fused with enhanced green fluorescent protein (EGFP) such that viral lytic reactivation can be monitored by flow cytometry (10). We transduced the reporter cells with a genome-wide CRISPR library and then induced KSHV lytic reactivation with doxycycline; the expression of the single guide RNA (sgRNA) library increased the percentage of GFP-positive cells during lytic reactivation (see Fig. S1 in the supplemental material). Fluorescence-associated cell sorting (FACS) was used to collect cells with the top 5% EGFP intensity (Fig. 1A). Cells that contain genetic alterations restricting KSHV lytic reactivation are expected to be enriched. sgRNA pools from sorted and input cells were amplified by PCR and quantified by next-generation sequencing. The MAGeCK algorithm was used to identify statistically significant hits (11), and we later focused on the most significantly enriched genes (Fig. 1B; Table S1). Among the hits, EED, a core subunit of PRC2, has been reported to repress lytic gene expression during KSHV *de novo* infection (12); IRF8 positively regulates STING-mediated innate immune responses to inhibit HSV-1 replication (13). The identification of known negative regulators of herpesvirus infection confirmed the reliability of our screening. To verify the screening results, we chose the top enriched hits and designed short hairpin RNA (shRNA) to knock down individual genes in KSHV latently infected cells. We found that depletion of SMCHD1, BAZ2B, MCTP1, PDCD10, NDUFB2, and GMPPA led to higher KSHV virion production (Fig. S1B), confirming that these host proteins function as restriction factors for KSHV lytic reactivation. In the next portion of our study, we focused on SMCHD1, as it was the most prominent hit, and we performed both shRNA-mediated knockdown and CRISPR-mediated knockout to validate the antiviral function of SMCHD1.

10.1128/mbio.00549-23.1 FIG S1 SMCHD1 is a potential restriction factor for KSHV lytic reactivation. (A) Cas9^+^ SLK.iBAC-ORF52-EGFP cells were transduced with the GeCKO v2 sgRNA library and induced with doxycycline to initiate lytic reactivation. Flow cytometry analysis was then performed to quantify the percentage of GFP-positive cells. (B) SLK.iBAC-GFP cells were transduced with control shRNA or shRNA targeting the indicated genes to generate stable cells. The stable cells were induced with Dox and sodium butyrate to trigger KSHV lytic replication. The supernatants were collected at 48 h postinduction and were used to infect 293T cells. KSHV viral titer was calculated based on flow cytometry analysis of GFP-positive cell percentages at 24 h postinfection. (C) The number of SLK.iBAC-GFP cells as described for panel D were quantified at the indicated time points. (D) SLK.iBAC-GFP cells were transduced with shRNA targeting SMCHD1 to generate knockdown cells. WCLs were analyzed by immunoblotting. (E) The number of BCBL-1 cells, as described for panel F, were quantified at the indicated time points. (F) BCBL-1 cells were transduced with sgRNA targeting SMCHD1 to generate knockout cells. WCLs were analyzed by immunoblotting. (G) Viral latent genes expression in the BCBL-1 stable cells as described for panel F were quantified by qRT-PCR. (H) The relative KSHV viral genomic copy number in the BCBL-1 stable cells as described for panel F was determined by qPCR. (I) The expression of viral latent genes in the SLK.iBAC-GFP stable cells as described for panel D were measured by qRT-PCR. Download FIG S1, TIF file, 0.5 MB. Copyright © 2023 Tian et al. 2023 Tian et al. https://creativecommons.org/licenses/by/4.0/ This content is distributed under the terms of the Creative Commons Attribution 4.0 International license.

10.1128/mbio.00549-23.6 TABLE S1 Summary of CRISPR screening results. Download Table S1, PDF file, 0.1 MB. Copyright © 2023 Tian et al. 2023 Tian et al. https://creativecommons.org/licenses/by/4.0/ This content is distributed under the terms of the Creative Commons Attribution 4.0 International license.

To confirm the antiviral activity of SMCHD1 against KSHV, we designed two independent sgRNAs that were different from those in the GeCKO library and abolished the expression of SMCHD1 in the reporter cells (Fig. 1C). Depletion of SMCHD1 greatly enhanced lytic reactivation of KSHV, as indicated by the enhanced EGFP intensity under a fluorescence microscope (Fig. 1D) and higher GFP-positive cell percentage as quantified by flow cytometry (Fig. 1E). Next, we wondered whether SMCHD1 affected KSHV latent infection. To test that, we turned to SLK.iBAC-GFP (SLK.iBAC, in short) cells that were latently infected with KSHV constitutively expressing EGFP and BCBL-1, a human primary effusion lymphoma (PEL) cell line that is naturally infected with KSHV. Depletion of SMCHD1 in SLK.iBAC or BCBL-1 cells did not affect cell proliferation (Fig. S1C and E). The expression of LANA, a viral latency marker, remained unchanged in SMCHD1-deficient cells, while RTA, a viral reactivation marker, was not induced upon SMCHD1 depletion (Fig. S1D and F). These data suggested that SMCHD1 was not involved in latency control. Furthermore, the transcription of viral latent genes and viral genome replication were comparable between wild-type and SMCHD1-depleted cells (Fig. S1G to I). These results ruled out the possibility that SMCHD1 contributes to latency control and the latent-to-lytic transition of KSHV.

Together, these data indicated that SMCHD1 is a restriction factor for KSHV lytic replication.

### SMCHD1 restricts KSHV lytic replication.

**FIG 2:**
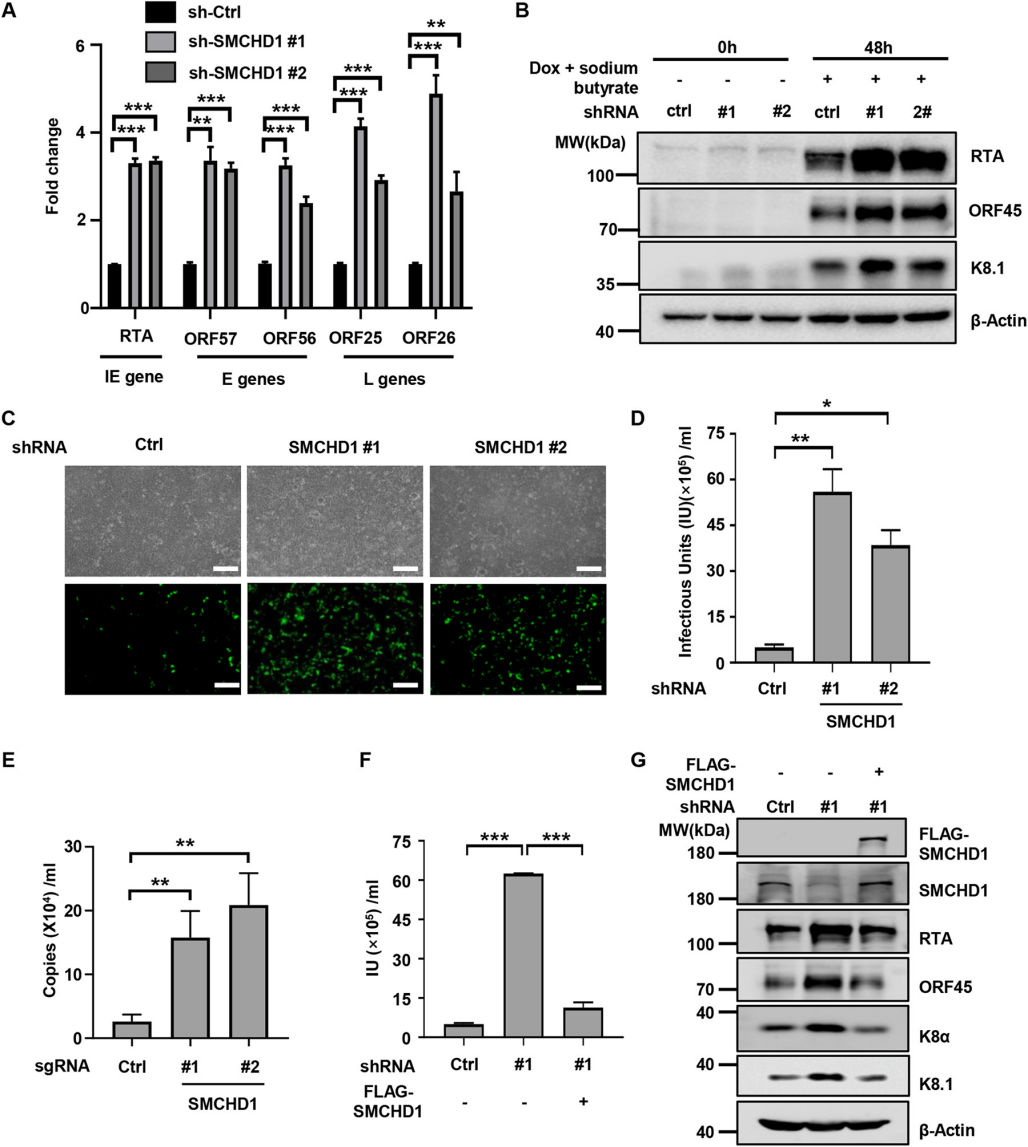
SMCHD1 restricts KSHV lytic reactivation. (A) SLK.iBAC-GFP cells were transduced with control shRNA or shRNA targeting SMCHD1 to generate stable cells. The stable cells were then induced with Dox, and viral gene expression was quantified by qRT-PCR at 48 h postinduction. (B) Immunoblot analysis of SLK.iBAC-GFP stable cells as described for panel A at the indicated time points postinduction. (C) SLK.iBAC-GFP stable cells, as described for panel A, were induced with Dox and sodium butyrate for 48 h. The supernatants were collected and used to infect 293T cells. GFP expression was imaged 24 h postinfection. Scale bars, 100 μm. (D) KSHV viral titer was calculated based on flow cytometry analysis of GFP-positive cell percentages as described for panel C. (E) BCBL-1-Tet-RTA cells were transduced with control sgRNA or sgRNA targeting SMCHD1, and the stable cells were induced by Dox for 96 h. KSHV viral copy numbers in the supernatants were quantified by qPCR. (F and G) SLK.iBAC-GFP SMCHD1 knockdown stable cells were stably reconstituted with vector control or SMCHD1 through lentiviral transduction. The reconstituted cells were induced with Dox to trigger lytic reactivation for 48 h. KSHV viral titers in the supernatants were quantified (F), and WCLs were analyzed by immunoblotting (G).

Next, we further characterized the antiviral activity of SMCHD1 against KSHV. Knockdown of SMCHD1 in SLK.iBAC cells significantly promoted the lytic replication of KSHV, as indicated by the enhanced transcription of an immediate early gene ORF50, early genes ORF57 and ORF56, and late genes ORF25 and ORF26 (Fig. 2A). Consistently, the protein levels of RTA, ORF45, and K8.1 were increased in SMCHD1-depleted cells (Fig. 2B). Moreover, silencing of SMCHD1 markedly enhanced the KSHV viral titer by ≈10-fold (Fig. 2C and D). Next, we used BCBL-1-Tet-RTA, a human PEL cell line that initiates lytic reactivation upon doxycycline induction, to validate that ablation of SMCHD1 greatly promoted KSHV lytic replication (Fig. S2A and B; Fig. 2E). Then, we asked whether ectopic expression of SMCHD1 suppressed KSHV replication. Indeed, SMCHD1 expression in SLK.iBAC cells suppressed KSHV replication and the expression of viral proteins (Fig. S2C to E). Finally, we performed rescue experiments to rule out the off-target effect of shRNA-mediated knockdown of SMCHD1. We reconstituted SMCHD1-depleted SLK.iBAC cells with an shRNA-resistant SMCHD1 construct. While knockdown of SMCHD1 markedly enhanced KSHV viral titer and viral protein abundance (RTA, ORF45, K8α, and K8.1), SMCHD1 reconstitution reversed the effect of SMCHD1 deficiency on KSHV lytic replication (Fig. 2F and G; Fig. S2F). Next, we asked whether SMCHD1 plays a role in KSHV *de novo* infection. Depletion of SMCHD1 in HUVEC, a primary endothelial cell line, enhanced the transcription of viral genes and viral genome replication during *de novo* infection (Fig. S2G to I), indicating that SMCHD1 restricts KSHV *de novo* infection. KSHV latency could still be established in SMCHD1-depleted HUVEC (data not shown), suggesting that although SMCHD1 suppresses viral replication during *de novo* infection, the disturbance is insufficient to disrupt KSHV latency establishment. Next, we sought to determine whether KSHV infection influences SMCHD1 protein level. We found that the SMCHD1 level was not affected during KSHV *de novo* infection (Fig. S2J). In contrast, the protein level of SMCHD1 was decreased during KSHV lytic reactivation in BCBL-1-Tet-RTA cells (Fig. S2K), suggesting that KSHV lytic reactivation may downregulate SMCHD1 to support viral replication in a feed-forward manner.

10.1128/mbio.00549-23.2 FIG S2 SMCHD1 restricts KSHV lytic replication. (A) BCBL-1-Tet-RTA cells were transduced with control sgRNA or sgRNA targeting SMCHD1 to generate knockout cells. The cells were then induced with Dox and sodium butyrate, and viral gene expression was quantified by qRT-PCR at 24 h postinduction. (B) BCBL-1-Tet-RTA cells were induced as described for panel A, and WCLs were collected at the indicated time points and applied to immunoblotting analysis. (C) SLK.iBAC-GFP cells stably transduced with a lentivirus that encoded control or SMCHD1 cDNA were induced with Dox and sodium butyrate. The supernatants were collected at 48 h postinduction and were used to infect 293T cells. GFP expression was imaged at 24 h postinfection. Scale bars, 100 μm. (D) KSHV viral titer was calculated based on flow cytometry analysis of GFP-positive 293T cell percentage as described for panel C. (E) SLK.iBAC-GFP stable cells were induced as described for panel C, and WCLs were collected at the indicated time points and applied to immunoblotting analysis. (F) SLK.iBAC-GFP SMCHD1 knockdown stable cells were reconstituted with vector control or SMCHD1 through lentiviral transduction. The reconstituted cells were induced with Dox and sodium butyrate to trigger lytic reactivation. The supernatants were collected at 48 h postinduction and were used to infect 293T cells. GFP expression was imaged at 24 h postinfection. Scale bars, 100 μm. (G) HUVEC cells were transduced with control sgRNA or sgRNA targeting SMCHD1 to generate knockout cells. WCLs were analyzed by immunoblotting. (H) qRT-PCR analysis of KSHV viral gene expression in KSHV-infected HUVEC stable cells at 12 h postinfection. (I) The relative KSHV viral genomic copy number in KSHV-infected HUVEC stable cells was determined by qPCR. (J) HUVEC cells were infected with KSHV. WCLs were collected at the indicated time points and analyzed by immunoblotting. (K) BCBL-1-Tet-RTA cells were induced with Dox to trigger lytic reactivation. WCLs were collected at the indicated time points and analyzed by immunoblotting. Download FIG S2, TIF file, 1.3 MB. Copyright © 2023 Tian et al. 2023 Tian et al. https://creativecommons.org/licenses/by/4.0/ This content is distributed under the terms of the Creative Commons Attribution 4.0 International license.

Collectively, these data indicated that SMCHD1 restricts KSHV lytic replication and *de novo* infection.

### SMCHD1 associates with KSHV ORI-Lyt and hampers KSHV genome replication.

**FIG 3:**
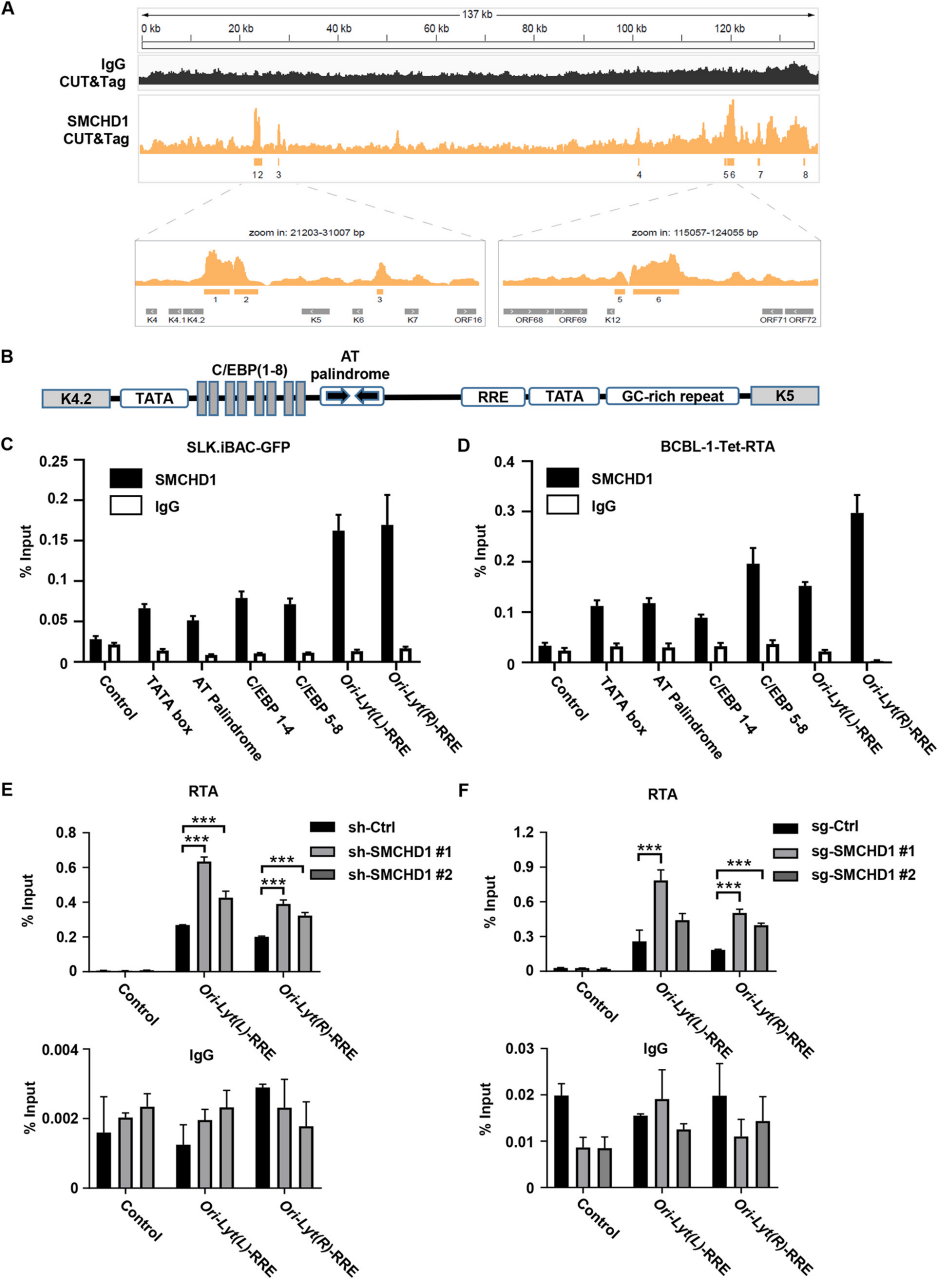
SMCHD1 binds to KSHV ORI-Lyt. (A) Genome-wide mapping of SMCHD1-binding sites on the viral genome during KSHV lytic replication by CUT&Tag. (B) Schematic diagram of KSHV Ori-Lyt(L). (C and D) SLK.iBAC-GFP or BCBL-1-Tet-RTA cells were treated with Dox to induce KSHV lytic reactivation. The binding of SMCHD1 to the indicated motifs of ORI-Lyt was determined by ChIP-qPCR assays at 24 h postinduction. The coding region of KSHV ORF20 served as a control. (E and F) SLK.iBAC-GFP (knockdown of SMCHD1) or BCBL-1-Tet-RTA cells (knockout of SMCHD1) were treated with Dox to induce KSHV lytic reactivation. The binding of RTA to the indicated motifs of ORI-Lyt was determined by ChIP-qPCR assays at 24 h postinduction. The coding region of KSHV ORF20 served as a control.

Recently, SMCHD1 has been shown to be weakly induced by chicken interferon α (14). Therefore, we investigated whether SMCHD1 could be induced by type I interferon. We found that *SMCHD1* was not induced by IFN-β in SLK.iBAC-GFP or BCBL-1 cells, whereas ISG56 was potently induced as a positive control (Fig. S3A). To confirm these results, we stimulated THP-1, a human monocytic cell line, with IFN-β and obtained similar results (Fig. S3A). Our data suggested that SMCHD1 is not an ISG. SMCHD1 is a well-characterized epigenetic regulator that directly associates with chromatin to repress transcription (15). Loss-of-function mutations of *SMCHD1* result in DNA hypomethylation of the *D4Z4* macrosatellite array on chromosome 4, which underlies the onset of facioscapulohumeral muscular dystrophy, a muscular developmental disease (8). The transcriptional repressor activity of SMCHD1 is also evident from its crucial role in X chromosome inactivation (8). We thus hypothesized that SMCHD1 may associate with the KSHV genome to restrict viral replication. To test this hypothesis, we performed cleavage under targets and tagmentation (CUT&Tag) assays to evaluate the chromatin profiling of SMCHD1 during KSHV lytic replication. Indeed, SMCHD1 was found to clearly associate with the KSHV genome; notably, the two main viral genomic regions occupied by SMCHD1 largely overlapped with the two origins of lytic DNA replication (ORI-Lyt) (Fig. 3A).

10.1128/mbio.00549-23.3 FIG S3 Knockdown of SMCHD1 promotes KSHV lytic replication. (A) BCBL-1-Tet-RTA, SLK.iBAC-GFP, and THP-1 cells were treated with IFN-β for 6 h. The expression of *SMCHD1* and *ISG56* was determined by qRT-PCR. (B) SLK.iBAC-GFP or BCBL1-Tet-RTA cells were transduced with the indicated shRNAs or sgRNAs to generate stable cells. The stable cells were induced with Dox, and KSHV intracellular genome copy number was quantified by qRT-PCR at 48 h postinduction. (C) SLK.iBAC-GFP control or SMCHD1 knockdown stable cells were transduced with FLAG-K8α through lentiviral transduction. The stable cells were induced with Dox to trigger lytic reactivation. The binding of FLAG-K8α to the indicated motifs of ORI-Lyt was determined by ChIP-qPCR at 24 h postinduction. (D) SLK.iBAC-GFP cells were treated with Dox to induce KSHV lytic reactivation. The binding of SMCHD1 to the indicated genes were determined by ChIP-qPCR at 24 h postinduction. The coding region of KSHV ORF20 served as a control. (E) SLK.iBAC-GFP control or SMCHD1 knockdown stable cells were treated with Dox to induce lytic reactivation. Genomic DNA was collected at 24 h postinduction and applied to bisulfite conversion followed by Sanger sequencing (8 in each group) to assess the methylation status of D4Z4 macrosatellite. Blue open circles denote methylated CpG dinucleotides, while red circles represent unmethylated CpG dinucleotides. The bottom row shows the percentage of DNA hypomethylation of CpG dinucleotides. (F) SLK.iBAC-GFP cells were treated with Dox to induce lytic reactivation. The transcription of DUX4 and of a viral gene, ORF56, were quantified by qRT-PCR at 24 h postinduction. Download FIG S3, TIF file, 1.2 MB. Copyright © 2023 Tian et al. 2023 Tian et al. https://creativecommons.org/licenses/by/4.0/ This content is distributed under the terms of the Creative Commons Attribution 4.0 International license.

KSHV contains two ORI-Lyts, and both ORI-Lyts share an almost identical core sequence, followed by GC-rich tandem repeats (16). Four motifs have been identified in the ORI-Lyt core sequence: TATA boxes, AT palindromic sequence, eight C/EBP-binding motifs, and an RTA responsive element (L-RRE and R-RRE) (Fig. 3B) (16). Chromatin immunoprecipitation (ChIP) analysis confirmed that SMCHD1 bound to these motifs of ORI-Lyt when KSHV lytic replication was induced in both SLK.iBAC and BCBL-1-Tet-RTA cells (Fig. 3C and D), indicating that SMCHD1 binding to KSHV ORI-Lyt was independent of cell type. Our CUT&Tag and ChIP data suggested that SMCHD1 binding to ORI-Lyt may block viral genome replication to inhibit KSHV lytic replication. Accordingly, depletion of SMCHD1 significantly increased KSHV genome copy number in both SLK.iBAC and BCBL-1-Tet-RTA cells upon lytic replication (Fig. S3B). To further explore how SMCHD1 targets ORI-Lyt to suppress KSHV genome replication, we assessed the recruitment of RTA and K8α to ORI-Lyt, which is essential for the docking of the viral replication complex onto ORI-Lyt and the subsequent initiation of viral genome replication (16). Remarkably, depletion of SMCHD1 in SLK.iBAC and BCBL-1-Tet-RTA cells significantly enhanced the association of RTA with both RREs during KSHV lytic replication (Fig. 3E and F). Similarly, the recruitment of K8α to ORI-Lyts was also greatly increased in SMCHD1-depleted cells (Fig. S3C). It is worth mentioning that K8α showed promiscuous binding rather than specific association at C/EBP-binding motifs in our experimental settings, which is consistent with previous reports (17, 18). Our CUT&Tag analysis revealed a small enrichment peak in the latency locus of KSHV, and ChIP analysis confirmed that SMCHD1 slightly but significantly associated with ORF71, ORF72, and ORF73 (Fig. S3D), suggesting that SMCHD1 may occupy other genomic regions of KSHV to restrict viral replication. Next, we asked whether KSHV reactivation led to DNA hypomethylation of the *D4Z4* macrosatellite. Bisulfite sequencing indicated that KSHV lytic reactivation did not affect the methylation of the D4Z4 macrosatellite in SLK.iBAC cells (Fig. S3E), consistent with the lack of induction of *DUX4* (Fig. S3F). These data suggested that KSHV lytic reactivation is insufficient to cause hypomethylation of the *D4Z4* macrosatellite.

Taken together, these results indicated that SMCHD1 binds to KSHV ORI-Lyt and interrupts the recruitment of RTA and K8 to ORI-Lyt to restrict KSHV genome replication.

### The hinge domain of SMCHD1 is required to restrict KSHV lytic replication.

**FIG 4:**
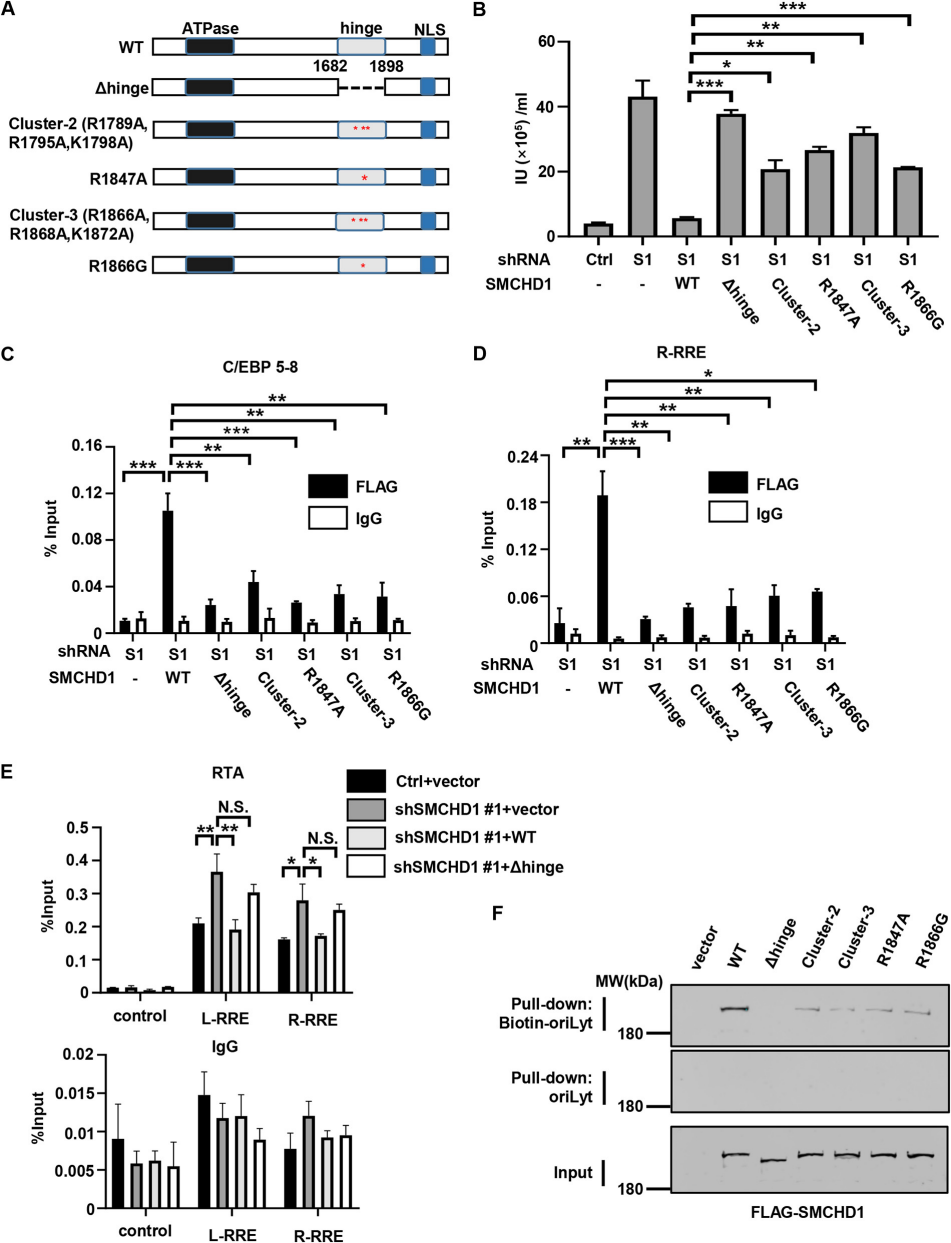
The hinge domain of SMCHD1 is required to restrict KSHV lytic replication. (A) Schematic diagram of SMCHD1 hinge domain mutants. (B) SMCHD1 stable knockdown SLK.iBAC-GFP cells were reconstituted with control vector (Ctrl), wild-type SMCHD1, or the indicated mutants (Δhinge, cluster-2, cluster-3, R1847A, or R1866G). The reconstituted cells were treated with Dox and sodium butyrate to induce KSHV lytic reactivation, and KSHV viral titers in the supernatants were quantified at 48 h postinduction. (C and D) The reconstituted cells, as described for panel B, were treated with Dox to induce KSHV lytic replication for 24 h. The binding of FLAG-SMCHD1 to the indicated motifs was determined by ChIP-qPCR assays. (E) SMCHD1 stable knockdown SLK.iBAC-GFP cells were reconstituted with control vector (Ctrl), wild-type SMCHD1, or the Δhinge mutant. The reconstituted cells were treated with Dox to induce KSHV lytic reactivation. The binding of RTA to the indicated motifs of ORI-Lyt was determined by ChIP-qPCR assays at 24 h postinduction. (F) Biotin-ORI-Lyt pulldown assay. WT SMCHD1 or the indicated hinge domain mutants were expressed in 293T cells. WCLs were collected and pulldown assays were performed by using biotin-ORI-Lyt or ORI-Lyt. The pulldown and input samples were analyzed by immunoblotting with FLAG antibody.

Previous reports indicated that the hinge domain of SMCHD1 has the capacity to bind DNA and mediates the direct interaction with chromatin (15, 19, 20). Therefore, we asked whether the hinge domain of SMCHD1 is required for its association with ORI-Lyt and whether and how the association contributes to the anti-KSHV activity of SMCHD1. To test that, we silenced endogenous SMCHD1 in SLK.iBAC cells and then reconstituted the SMCHD1-depleted cells with wild-type (WT) SMCHD1, Δhinge mutant (with amino acids [aa] 1682 to 1898 deleted; Δ1682-1898), or a series of mutants that were previously reported to have impaired DNA-binding activity {cluster 2 (Arg-to-Ala change at position 1789 [R1789A], R1795A, and K1798A), cluster 3 [R1866A, R1868A, and K1872A], R1847A, and R1866G} by lentiviral transduction (15, 21) (Fig. 4A and Fig. S4A). While WT SMCHD1 reconstitution consistently suppressed KSHV lytic replication, the Δhinge mutant was completely defective for restriction of KSHV replication, and the other four mutants all showed compromised restrictive activity (Fig. 4B). These results demonstrated that the hinge domain of SMCHD1 is required to restrict KSHV lytic replication and strongly suggested that the DNA-binding activity of the hinge domain is necessary for the antiviral activity of SMCHD1. Next, we sought to evaluate whether the hinge domain of SMCHD1 is required to associate with KSHV ORI-Lyt. First, we took advantage of the reconstituted SLK.iBAC cells that expressed WT SMCHD1 or the mutants to compare their binding capacity with ORI-Lyt using ChIP assays. Indeed, depletion of the hinge domain nearly abolished ORI-Lyt association, and the other four mutants (cluster 2, cluster 3, R1847A, and R1866G) showed significantly reduced ORI-Lyt binding toward all the motifs of ORI-Lyt (R-RRE, L-RRE, C/EBP, AT palindrome, and TATA box) (Fig. 4C and D, Fig. S4B to E). Consistently, WT SMCHD1 but not the Δhinge mutant blocked the recruitment of RTA and K8α to ORI-Lyt (Fig. 4E and Fig. S4F). We further evaluated the interaction between the hinge domain of SMCHD1 with ORI-Lyt by an *in vitro* pulldown assay using recombinant proteins purified from bacteria. Purified hinge domain efficiently associated with ORI-Lyt, while it did not bind to control DNA segments, such as a nonrelevant KSHV genome sequence and the coding sequence of EGFP (Fig. S4G), indicating that the hinge domain specifically associates with ORI-Lyt. Since full-length SMCHD1 could not be successfully expressed and purified from bacteria (20, 21), we transiently expressed SMCHD1 or its mutants in HEK293T cells and performed pulldown experiments. Again, WT SMCHD1 robustly interacted with ORI-Lyt, and the Δhinge mutant was defective in ORI-Lyt association; the other four mutants demonstrated markedly reduced binding activity (Fig. 4F). Our results suggested that SMCHD1 binds to ORI-Lyt and blocks the recruitment of RTA and K8α through steric hindrance. To validate this conclusion, we employed sgRNA to target an endonuclease-dead Cas9 mutant (dCas9) to the ORI-Lyt binding site of SMCHD1. We found that the targeting of dCas9 at the ORI-Lyt potently suppressed KSHV lytic replication (Fig. S4H and I), even though dCas9 (~160 kDa) was considerably smaller than SMCHD1 (~226 kDa). These data supported a model whereby SMCHD1 associates with ORI-Lyt and restricts KSHV lytic replication through steric hindrance.

10.1128/mbio.00549-23.4 FIG S4 The hinge domain of SMCHD1 is required to restrict KSHV lytic replication. (A) SMCHD1 stable knockdown SLK.iBAC-GFP cells were reconstituted with control vector (Ctrl), wild-type SMCHD1, or the indicated mutants (Δhinge, cluster-2, cluster-3, R1847A, R1866G). WCLs were analyzed by immunoblotting. (B to E) The reconstituted cells, as described for panel A, were treated with Dox to induce KSHV lytic replication. The binding of FLAG-SMCHD1 to the indicated motifs was determined by ChIP-qPCR assays at 24 h postinduction. (F) SMCHD1 stable knockdown SLK.iBAC-GFP cells were reconstituted with control vector (Ctrl), wild-type SMCHD1, or Δhinge mutant. The reconstituted cells were transduced with HA-K8α through lentiviral transduction. The stable cells were treated with Dox to induce KSHV lytic reactivation. The binding of HA-K8α to the indicated motifs of ORI-Lyt was determined by ChIP-PCR assays at 24 h postinduction. (G) Biotin-ORI-Lyt pulldown assay. SMCHD1 hinge domain (aa 1682 to 1898) was expressed and purified from E. coli. Pulldown assays were performed by using biotin-ORI-Lyt or control (biotin-ORF21 and biotin-EGFP). The pulldown and input samples were analyzed by immunoblotting with 6×His antibody. (H) SMCHD1 stable knockdown SLK.iBAC-GFP cells were transduced with control sgRNA or sgRNA targeting the ORI-Lyt binding site of SMCHD1. WCLs were analyzed by immunoblotting. (I) The stable cells as described for panel H were treated with Dox and sodium butyrate to induce KSHV lytic reactivation. The supernatants were collected and were used to infect with 293T cells. KSHV viral titer was calculated based on flow cytometry analysis of GFP-positive 293T cell percentage. Download FIG S4, TIF file, 0.6 MB. Copyright © 2023 Tian et al. 2023 Tian et al. https://creativecommons.org/licenses/by/4.0/ This content is distributed under the terms of the Creative Commons Attribution 4.0 International license.

Collectively, these data indicated that SMCHD1 associates with KSHV ORI-Lyt through the hinge domain to restrict KSHV lytic replication.

### SMCHD1 restricts replication of a broad range of herpesviruses.

**FIG 5:**
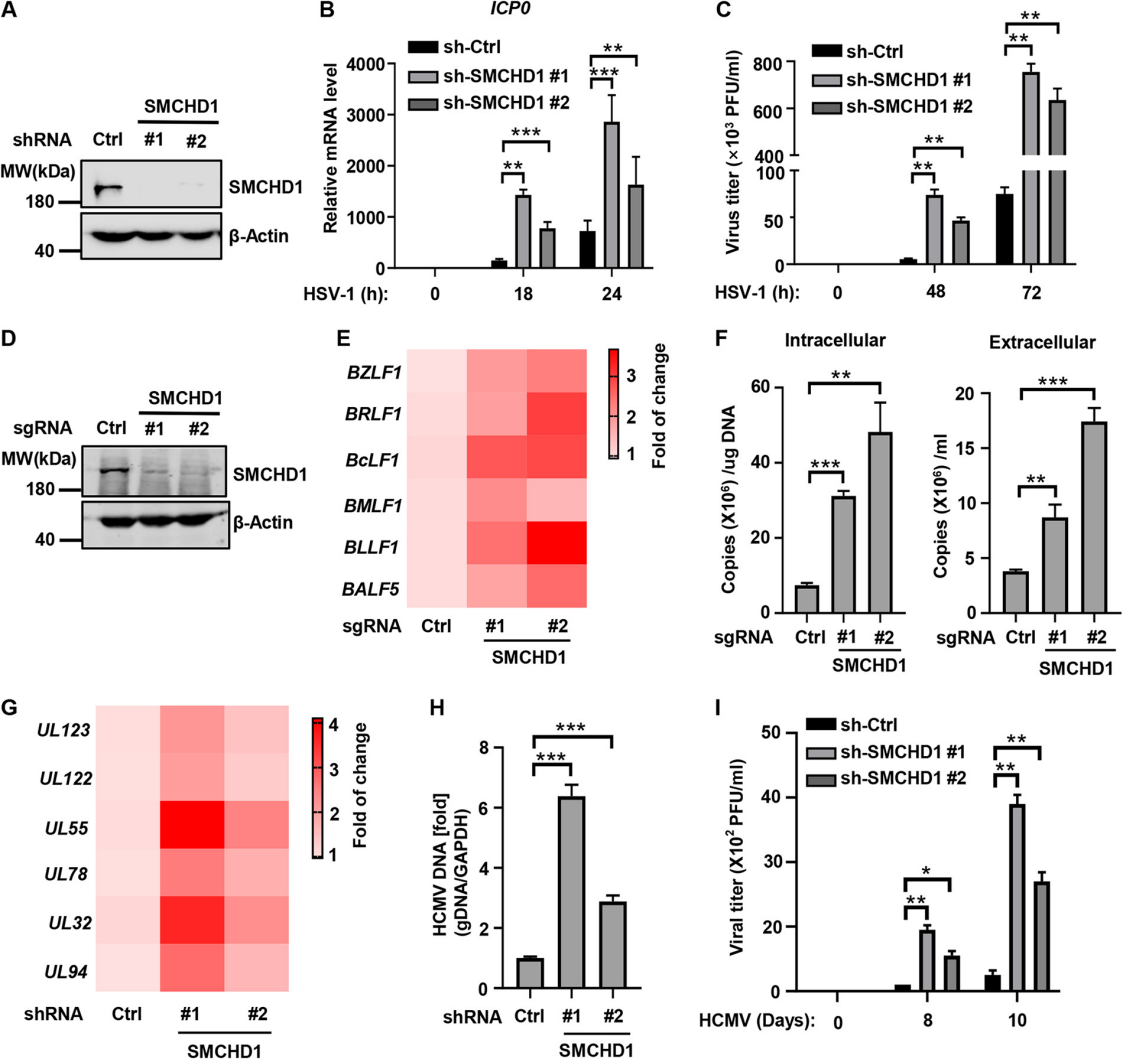
SMCHD1 restricts the replication of diverse herpesviruses. (A) U2OS cells were transduced with control shRNA or shRNA targeting SMCHD1 to generate stable cells, and WCLs were applied to immunoblotting analysis. (B and C) Control (sh-Ctrl) or SMCHD1 stable knockdown (sh-SMCHD1) U2OS cells as described for panel A were infected with HSV-1 (MOI, 0.1). The expression of the viral gene (*ICP0*) was determined by RT-qPCR at the indicated time points postinfection. HSV-1 viral titers were measured by standard plaque assay at the indicated time points postinfection. (D) P3HR-1 cells were transduced with control sgRNA or sgRNA targeting SMCHD1, and WCLs were collected for immunoblot analysis with the indicated antibodies. (E and F) P3HR-1 stable cells as described for panel D were induced with PMA and sodium butyrate for 48 h. The heatmap summarizes the relative expression of EBV viral genes determined by qRT-PCR (E); the intracellular and extracellular EBV genomic copies were quantified by qPCR analysis (F). (G) Human foreskin fibroblasts (HFF) cells were transduced with control shRNA or shRNA targeting SMCHD1, and the HFF stable cells were infected with HCMV-GFP (MOI, 0.01) for 48 h. The heatmap summarizes the relative expression of HCMV viral genes determined by qRT-PCR. (H) HFF control or SMCHD1 knockdown stable cells were infected with HCMV-GFP (MOI, 0.1) for 96 h. The relative HCMV viral genomic copy number was determined by qPCR. (I) HFF control or SMCHD1 knockdown stable cells were infected with HCMV-GFP (MOI, 0.01), and viral titer at the indicated time points was determined by plaque assays using HFF cells.

Herpesvirus genomes consist of 1 to 3 *cis*-acting sites that serve as origins of lytic DNA replication. Regardless of the diverse herpesvirus types, a set of six viral proteins that are conserved across the *Herpesviridae* family form an enzymatic complex that is then recruited to ORI-Lyt to initiate viral genome replication (1, 16). The conserved replication strategy employed by *Herpesviride* prompted us to propose that SMCHD1 may restrict a broad range of herpesviruses. In support of this hypothesis, silencing SMCHD1 significantly enhanced gene expression of HSV-1, a model alphaherpesvirus (Fig. 5A and B; Fig. S5A). Accordingly, viral titers in the supernatants of SMCHD1-depleted cells were 8- to 13-fold higher than in control cells (Fig. 5C). Depletion of SMCHD1 also greatly promoted the replication of Epstein-Barr virus (EBV), another gammaherpesvirus closely related to KSHV, as demonstrated by the substantially increased viral gene expression in SMCHD1-depleted cells (Fig. 5D and E; Fig. S5B). Moreover, the intracellular and extracellular EBV genome copy number were markedly enhanced in SMCHD1-depleted cells (Fig. 5F). Furthermore, ablation of SMCHD1 also led to enhanced lytic replication of human cytomegalovirus (HCMV), a human betaherpesvirus, as indicated by the enhanced viral gene expression, increased viral genome replication, and infectious viral titer (Fig. S5C; Fig. 5G to I). Finally, sgRNA-mediated depletion of SMCHD1 in both mouse and human cells promoted the replication of mouse herpesvirus 68 (MHV68), a murine gammaherpesvirus closely related to KSHV and EBV, as demonstrated by a marked enhanced viral titer in the supernatant of SMCHD1-depleted cells compared with those from control cells (Fig. S5D to G). Consistently, SMCHD1 efficiently associated with ORI-Lyts of HSV-1, HCMV, and MHV68 (Fig. S5H). These data collectively indicated that SMCHD1 is a pan-herpesvirus restriction factor that efficiently restrict a broad range of herpesviruses.

10.1128/mbio.00549-23.5 FIG S5 SMCHD1 restricts the replication of diverse herpesviruses. (A) U2OS cells were transduced with control shRNA or shRNA targeting SMCHD1 to generate stable cells. Control (sh-Ctrl) or SMCHD1 stable knockdown U2OS cells were infected with HSV-1 (MOI, 0.1). The expression levels of the viral genes (*ICP34.5* and *UL23*) were determined by RT-qPCR at the indicated time points postinfection. (B) P3HR-1 stable cells as described for panel D were induced with PMA and sodium butyrate for 24 h. The heatmap summarizes the relative expression of EBV viral genes determined by qRT-PCR. (C) HFF cells were transduced with control shRNA or shRNA targeting SMCHD1 to generate stable cells, and WCLs were applied to immunoblotting analysis. (D and E) NIH 3T3 or A549 cells were transduced with control sgRNA or sgRNA targeting SMCHD1 to generate stable cells, and WCLs were analyzed by immunoblotting. (F) NIH 3T3 stable cells as described for panel D were infected with MHV68-GFP (MOI, 0.1), and the viral titer at the indicated time points was determined by plaque assay using BHK21 cells. (G) A549 stable cells as described for panel E were infected with MHV68-GFP (MOI, 0.1). Viral titer at the indicated time points was determined by plaque assays using BHK21 cells. (H) Biotin-Ori-Lyt pulldown assay. FLAG-SMCHD1 was expressed in 293T cells and WCLs were prepared. Biotin-Ori-Lyt pulldown assays were performed by using biotin labeled-EGFP, HSV-1 Ori-Lyt, HCMV Ori-Lyt, or MHV68 Ori-Lyt. The pulldown and input samples were analyzed by immunoblotting with FLAG antibody. Download FIG S5, TIF file, 0.4 MB. Copyright © 2023 Tian et al. 2023 Tian et al. https://creativecommons.org/licenses/by/4.0/ This content is distributed under the terms of the Creative Commons Attribution 4.0 International license.

### SMCHD1 deficiency facilitated MHV68 replication *in vivo*.

**FIG 6:**
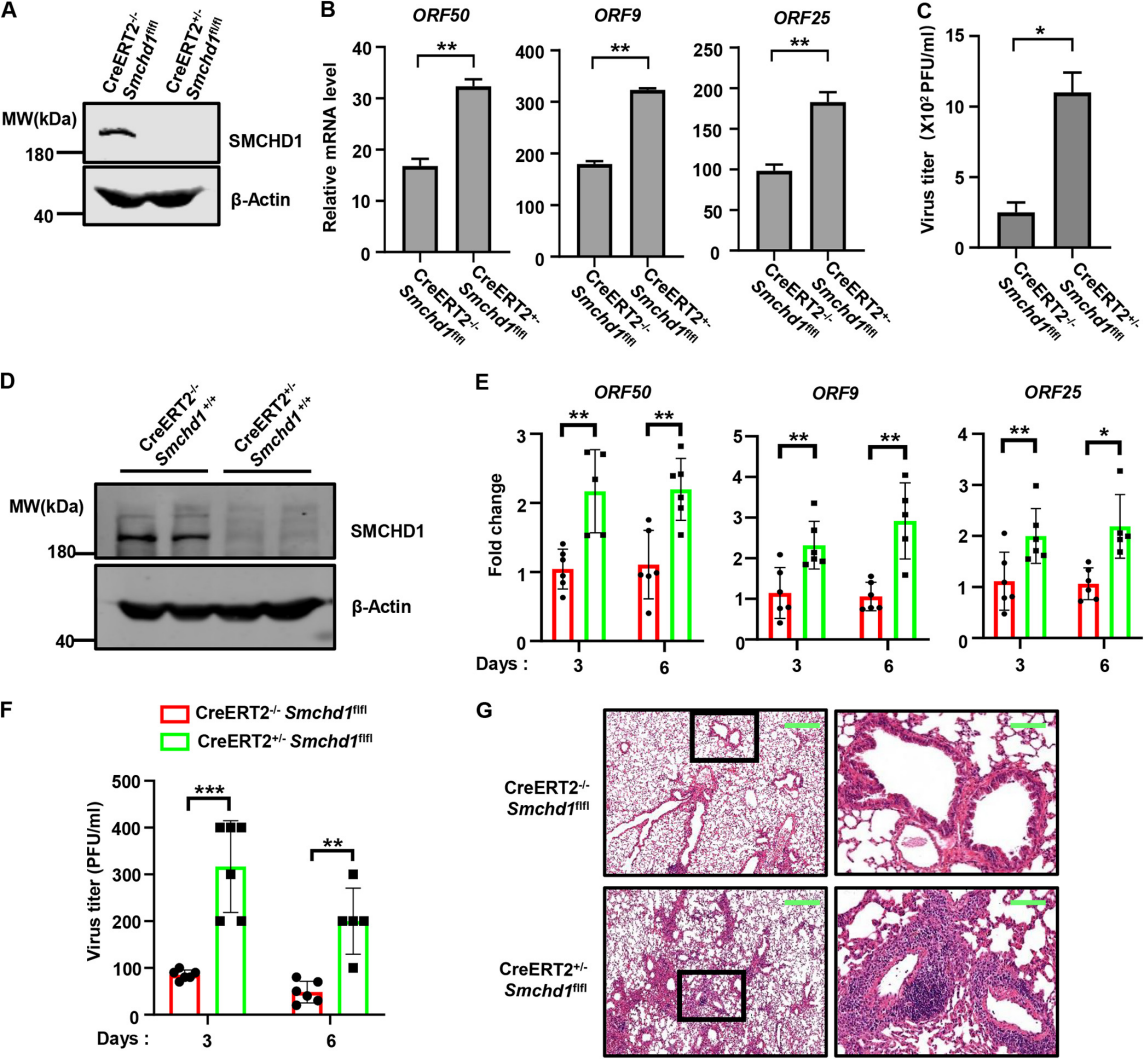
SMCHD1 deficiency facilitates MHV68 replication *in vivo*. (A) MLFs derived from Cre-ERT2^−/−^ *Smchd1*^fl/fl^ and Cre-ERT2^+/−^ *Smchd1*^fl/fl^ mice were treated with 4-hydroxytamoxifen (1 μM) for 3 days to generate knockout cells. WCLs were prepared and analyzed by immunoblotting. (B) SMCHD1 knockout MLFs as described for panel A were infected with MHV68 (MOI, 0.1), and viral gene expression was quantified by qRT-PCR at 48 h postinfection. (C) SMCHD1 knockout MLFs as described for panel A were infected with MHV68 (MOI, 0.01), and viral titer was determined at 48 h postinfection. (D) Cre-ERT2^−/−^ *Smchd1*^fl/fl^ and Cre-ERT2^+/−^ *Smchd1*^fl/fl^ mice were intraperitoneally injected with tamoxifen. The protein level of SMCHD1 in the lungs was analyzed by immunoblotting at 3 days postinfection. (E and F) Cre-ERT2^−/−^ *Smchd1*^fl/fl^ and Cre-ERT2^+/−^ *Smchd1*^fl/fl^ mice were intranasally infected with MHV68. Viral gene expression and viral titer in the lungs were quantified at 3 and 6 days postinfection. (G) Hematoxylin and eosin-stained mouse lung tissue sections 3 days postinfection. Scale bars, 600 μm (left) and 100 μm (right).

Murine herpesvirus MHV68 readily infects laboratory mice, and its infection leads to acute replication lasting around 2 weeks, providing a valuable model to investigate herpesvirus replication *in vivo* (22). As germline depletion of SMCHD1 results in female-specific lethality (23), we generated *Smchd1*^fl/fl^ mice and then crossed them with ROSA26-CreERT2 mice to obtain Cre-ERT2^−/−^ *Smchd1*^fl/fl^ and Cre-ERT2^+/−^ *Smchd1*^fl/fl^ mice. First, we generated primary mouse lung fibroblasts (MLFs) from these mice and induced SMCHD1 knockout with 4-hydroxytamoxifen (Fig. 6A). Consist with our previous results, MHV68 replication was much higher in SMCHD1 knockout MLFs compared with control cells, as indicated by the enhanced viral gene expression and increased viral titer (Fig. 6B and C). To characterize the role of SMCHD1 in MHV68 replication *in vivo*, we administered tamoxifen to Cre-ERT2^−/−^ *Smchd1*^fl/fl^ and Cre-ERT2^+/−^ *Smchd1*^fl/fl^ mice and then infected the mice with MHV68 via intranasal inoculation. As expected, tamoxifen treatment depleted SMCHD1 in the lungs of Cre-ERT2^+/−^ *Smchd1*^fl/fl^ mice but not in Cre-ERT2^−/−^ *Smchd1*^fl/fl^ mice (Fig. 6D). The expression of multiple viral genes (ORF50, ORF9, and ORF25) was significantly higher in lungs of SMCHD1-deficient mice than in those of control mice (Fig. 6E). Plaque assays corroborated that SMCHD1 deficiency led to significantly increased MHV68 viral titer in the lung tissue (Fig. 6F). Consistently, the lungs of SMCHD1-deficient mice showed elevated inflammation and more infiltration of immune cells than did those of control mice after MHV68 infection (Fig. 6G). These data collectedly indicated that SMCHD1 deficiency facilitates MHV68 replication *in vivo*.

## DISCUSSION

In this study, we identified SMCHD1 as a restriction factor for KSHV lytic replication through a genome-wide CRISPR-Cas9 knockout screen. To reveal the mechanism by which SMCHD1 suppresses KSHV replication, we employed CUT&Tag assays to assess the SMCHD1-DNA interaction on a genome-wide scale (24). Remarkably, SMCHD1 mainly occupies the two origins of lytic DNA replication (ORI-Lyts) of KSHV, suggesting that SMCHD1 primarily inhibits virus genome replication. Along this line, the DNA-binding activity of the hinge domain of SMCHD1 is required for the targeting of ORI-Lyt and the antiviral activity against KSHV. SMCHD1 potently suppresses a wide range of herpesviruses, including alpha, beta, and gamma subfamilies, and thus functions as a pan-herpesvirus restriction factor.

Previously, genome-wide CRISPR screens have been performed to identify host factors required for survival and proliferation of KSHV latently infected cells or KSHV-transformed primary rat mesenchymal embryonic stem cells (25, 26). Multiple proteins related to mitochondrial translation and a nuclear export receptor XPO1 were identified in these screens, providing potential therapeutic targets for the treatment of KSHV-associated malignancies (25, 26). In this study, genome-wide CRISPR screening was performed to interrogate KSHV lytic reactivation. The characterization of SMCHD1 as a restriction factor provided valuable insights into host factors that modulate KSHV lytic replication, which contributes significantly to KSHV-induced oncogenesis through promoting virus spread and the secretion of proangiogenic and proinflammatory factors (27–29). How the other host factors identified in our screen regulate KSHV lytic reactivation and whether and how they contribute to KSHV-related malignances warrant further study.

To guard against pathogens, including herpesviruses, mammalian hosts deploy a large number of protein-based antiviral effectors. These effectors can be roughly divided into two groups: interferon-induced effectors and cell-intrinsic effectors. It is well-recognized that ISG-encoded antiviral effectors restrict the replication of diverse viruses, including herpesviruses (2, 4). For example, cGAS is induced by type I interferons and functions as a cytosolic DNA sensor that activates the type I interferon pathway to inhibit the replication of a broad range of herpesviruses, including HSV-1, HCMV, and KSHV (30–34). Interferon-induced MxB is a potent human herpesvirus restriction factor that blocks the uncoating of viral DNA from the incoming viral capsid and suppresses viral gene expression (5, 6). TRIM22 inhibits HSV-1 replication by epigenetic silencing of immediate-early genes and also potently restricts the replication of HCMV and EBV (35). The IFITs are broadly induced during KSHV lytic reactivation in epithelial cells, and they positively contribute to the induction of interferons and ISGs to restrict KSHV replication (7). Recently, it has become increasingly appreciated that cell-intrinsic effectors install a frontline defense against invading pathogens prior to the induction of interferons and ISGs. For example, centrosomal protein TRIM43 is robustly induced by herpesvirus infection (but not interferons) and potently restricts a broad range of herpesviruses by regulating nuclear lamina integrity (36). Our study uncovered SMCHD1 as an intrinsic restriction factor for herpesviruses which targets viral origins of replication (ORIs) to inhibit viral replication. Interestingly, a recent study showed that SMCHD1 is weakly induced by chicken interferon α and suppresses the transcription of duck hepatitis B virus covalently closed circular DNA (14). However, the abundance of SMCHD1 was not altered by interferon treatment in our experiment settings. Whether these discrepancies reflect species-specific responses to interferon stimulation remains to be explored.

It is well established that herpesviruses exploits host epigenetic machineries to control the switch between two life cycles, latency and lytic reactivation (37, 38). Previous studies revealed that EZH2-mediated H3K27me3 histone modification is required for the maintenance of KSHV latency and that the inhibition or deletion of EZH2 leads to KSHV lytic reactivation (39, 40). Moreover, KSHV infection resolves the bivalent chromatin of the PROX1 gene to promote the mesenchymal-to-endothelial transition (41). SMCHD1 is a well-characterized epigenetic regulator that directly associates with chromatin to repress transcription (15). Considering that SMCHD1 also associates with KSHV genome regions other than ORI-Lyt, SMCHD1 may play a role in altering KSHV chromatin epigenetic structure to affect both viral genome replication and gene expression. Moreover, SMCHD1 protein level was decreased during KSHV lytic reactivation, suggesting that KSHV lytic reactivation downregulates SMCHD1 to support viral replication in a feed-forward manner.

It is well established that SMCHD1 possesses DNA-binding activity, and the hinge domain of SMCHD1 binds DNA directly (15, 19). Previous ChIP-seq and *in vitro* binding assays indicated that SMCHD1 preferentially binds poly(dC)/poly(dG) rather than poly(dA) (15). However, the exact binding mode of SMCHD1 remains unclear. We have shown here that SMCHD1 binds to ORI-Lyts of diverse herpesviruses, although sequence alignments showed no apparent homology between these ORI-Lyts (data not shown). We reasoned that poly(dC)/poly(dG) preference could not explain the binding specificity of SMCHD1, since the whole KSHV genome has a high GC content. Considering that the KSHV core replication complex can efficiently replicate EBV ORI-Lyt (42), it is tempting to speculate that the conformation and/or functionality of ORI-Lyt may contribute to SMCHD1 association. Moreover, we cannot rule out the possibility that the association of SMCHD1 with a viral genomic region other than ORI-Lyt or host chromatin may contribute partly to its antiviral activity, although our data indicate that SMCHD1 preferentially targets the ORI-Lyt of KSHV.

In summary, SMCHD1 has been identified as a restriction factor for KSHV lytic replication through genome-wide CRISPR-Cas9 knockout screening. SMCHD1 associates with KSHV ORI-Lyt depending on the DNA-binding activity of its hinge domain. SMCHD1 deficiency facilitates the replication of a murine herpesvirus *in vivo*. The characterization of SMCHD1 as a pan-herpesvirus restriction factor deepens our understanding of the arms race between herpesviruses and host, which could be harnessed to develop new therapeutics for the treatment of herpesvirus infection and related diseases.

## MATERIALS AND METHODS

### Mice.

The *Smchd1*^fl/+^ mice were generated by Cyagen Biosiences (Guangzhou, China) through CRISPR/Cas9-mediated genome engineering. ROSA26-CreERT2 mice were kindly provided by Bo Zhong (43). The *Smchd1*^fl/fl^ mice were crossed with ROSA26-CreERT2 to obtain Cre-ERT2^−/−^ *Smchd1*^fl/fl^ and Cre-ERT2^+/−^ *Smchd1*^fl/fl^ mice.

To knock out SMCHD1 in primary MLFs derived from Cre-ERT2^−/−^ *Smchd1*^fl/fl^ and Cre-ERT2^+/−^ *Smchd1*^fl/fl^ mice, MLFs were treated with 4-hydroxytamoxifen (1 μM; Sigma catalog number H6278) for 3 days. To deplete SMCHD1 *in vivo*, 6- to 8-week-old Cre-ERT2^−/−^ *Smchd1*^fl/fl^ and Cre-ERT2^+/−^ *Smchd1*^fl/fl^ mice were injected with tamoxifen (intraperitoneally, 80 μg/g of body weight, dissolved in corn oil; MCE, HY-13757A) for five consecutive days. After another 7 days, the mice were inoculated intranasally with MHV68 (400 PFU). The lungs of the infected mice were collected at 3 and 6 days postinfection, and viral gene expression and viral titer were quantified by reverse transcription-quantitative PCVR (qRT-PCR) and plaque assay.

### Cell culture.

U2OS, HEK293T, VERO, HUVEC, NIH 3T3 (ATCC), and BHK21 cells (kindly provided by Hongyu Deng, Institute of Biophysics, Chinese Academy of Sciences) were cultured in Dulbecco’s modified Eagle medium (DMEM; Sigma) supplemented with 10% fetal calf serum (FCS; CellMax, Beijing, China) and 1% penicillin-streptomycin (HyClone). The human SLK cells carrying KSHV clone iBAC (SLK.iBAC-GFP and SLK.iBAC-ORF52-EGFP) were kindly provided by Fanxiu Zhu (Florida State University) (9) and were cultured in DMEM supplemented with 10% FCS and hygromycin B (500 μg/mL). To induce lytic reactivation, SLK.iBAC-GFP cells were treated with doxycycline (Dox; 1 μg/mL) and sodium butyrate (0.1 mM). BCBL-1 cells were kindly provided by Ke Lan (Wuhan University). BCBL-1-Tet-RTA cells were generated by stably transducing BCBL-1 with a doxycycline-inducible RTA construct. To induce KSHV reactivation, BCBL-1-Tet-RTA cells were treated with Dox (1 μg/mL) and sodium butyrate (0.2 mM). P3HR-1 cells were kindly provided by Yan Wang (Sun Yat-Sen University) and Yan Yuan (University of Pennsylvania) (44). To induce EBV reactivation, P3HR-1 cells were treated with phorbol 12-myristate 13-acetate (PMA; 25 ng/mL) and sodium butyrate (0.2 mM). BCBL-1 and P3HR-1 cells were maintained in RPMI 1640 (HyClone) supplemented with 10% FCS and penicillin-streptomycin. Primary human foreskin fibroblasts (HFF) were kindly provided by Bo Zhong (Wuhan University) and Min-Hua Luo (Wuhan Institute of Virology), and HFFs were grown in DMEM supplemented with 10% FCS and 1% penicillin-streptomycin (45). Primary MLFs were generated and cultured as previously described (45).

### Viruses.

HSV-1 was propagated using Vero cells, and virus titers were measured by standard plaque assay using Vero cells (45). HCMV-GFP was kindly provided by Hong-Bing Shu and Ming-Ming Hu (Wuhan University) and was propagated and titrated using HFF cells (30, 46). MHV68 and MHV68-GFP were kindly provided by Hongyu Deng (Institute of Biophysics, Chinese Academy of Sciences) and were propagated and titrated using BHK21 cells as previously described (47, 48). KSHV virions were collected from the culture supernatant of Dox-induced (1 μg/mL) and sodium butyrate-induced (0.1 mM) SLK.iBAC-GFP cells and used to infect HEK293T cells. KSHV viral titer was calculated based on flow cytometry analysis of the GFP-positive HEK293T cell percentage, which was used to calculate viral titer, in infectious units (IU) per milliliter.

### Antibodies and regents.

Commercial antibodies used in this study included mouse monoclonal FLAG (catalog number 2064, Dia-An Biotechnology [Wuhan, China]; 1:10,000), mouse monoclonal β-actin (catalog number 2060 Dia-An Biotechnology; 1:5,000); mouse monoclonal LANA (1:1,000) and rabbit polyclonal RTA (1:1,000) (both gifts from Ke Lan); KSHV ORF45 (catalog number sc-53883; 1:1,000), KSHV K8.1A/B antibody (catalog number sc-65446; 1:1,000), and K8α antibody (catalog number sc-69797; 1:1,000) were obtained from Santa Cruz Biotechnology; mouse monoclonal 6×His (66005-1-Ig; 1:3,000; Proteintech, Wuhan, China); IRDye 800CW goat anti-rabbit and IRDye 800CW goat anti-mouse (both at 1:10,000; both from Li-Cor); and goat anti-rabbit (H+L) horseradish peroxidase (HRP) conjugate and goat anti-mouse (H+L)-HRP conjugate (both at 1:10,000) were both from Bio-Rad.

Rabbit polyclonal anti-SMCHD1 was raised against recombinant human SMCHD1 (aa 110 to 600) by Dia-An Biotechnology (Wuhan, China) and was used at a 1:500 dilution.

Biotin-labeled ORI-Lyt DNA was ordered from Sangon Biotech (Shanghai, China). Formaldehyde, doxycycline, and sodium butyrate were ordered from Sigma-Aldrich. Puromycin, hygromycin B, blasticidin, and G418 were ordered from Invivogen. PMA was ordered from MedChemExpress.

### Plasmids.

SMCHD1 was amplified from cDNA and subcloned into pCDH-CMV-EF1α-Puro and pCDH-CMV-EF1α-NEO (System Biosciences) with a 3× Flag tag. For the rescue experiments, SMCHD1 with synonymous mutations at the shRNA (#1) targeting site was constructed into pCDH-CMV-EF1α-NEO. The SMCHD1 mutants [Δhinge (Δ1682-1898); cluster 2 (R1789A, R1795A, K1798A); R1847A; cluster 3 (R1866A, R1868A, K1872A); R1866G] were generated via site-directed mutagenesis and were constructed into pCDH-CMV-EF1α-NEO. K8α was amplified from cDNA and subcloned into pCDH-CMV-EF1α-Puro. BZLF1 was amplified from cDNA and subcloned into pEF-EF1α-FLAG-N vector. ORF50/RTA was amplified from KSHV BAC16 (49) and subcloned into pLVX-TetOne (TaKaRa Bio) and pEGFP-C1. The SMCHD1 hinge domain (aa 1682 to 1898) were constructed into pET-28a. All plasmids were verified by Sanger sequencing.

### RNA extraction and qRT-PCR.

SLK.iBAC-GFP cells were treated with Dox (1 μg/mL); BCBL-1-Tet-RTA cells were treated with Dox (1 μg/mL) and sodium butyrate (0.2 mM); P3HR-1 cells were induced with PMA (25 ng/mL) and sodium butyrate (0.2 mM); HFFs and U2OS cells were infected with HCMV-GFP (multiplicity of infection [MOI], 0.1) or HSV-1 (MOI, 1), respectively.

Total RNA was extracted using TRIzol reagent (TaKaRa) at the indicated time points postinduction or postinfection. One microgram of total RNA was used for reverse transcription with a HiScript II 1st Strand cDNA synthesis kit (Vazyme, Nanjing, China) according to the manufacturer’s instructions. The cDNA mixture was diluted 40 times and was then subjected to qRT-PCR analysis with SYBR green qPCR master mix (Bimake, Shanghai, China). The relative expression levels of the targets genes were normalized to the expression of *ATCB*.

The primer sequences for qRT-PCR are provided in Table S2.

10.1128/mbio.00549-23.7 TABLE S2 Primers used in the study. Download Table S2, PDF file, 0.2 MB. Copyright © 2023 Tian et al. 2023 Tian et al. https://creativecommons.org/licenses/by/4.0/ This content is distributed under the terms of the Creative Commons Attribution 4.0 International license.

### Genome-wide CRISPR-Cas9 knockout screen.

The screening workflow is illustrated in Fig. 1A. We generated Cas9-expressing SLK.iBAC-ORF52-EGFP stable cells by transducing a Cas9 coding construct (Addgene catalog number 52962). The Cas9^+^ SLK.iBAC-ORF52-EGFP cells were then transduced with the GeCKO v2.0 human CRISPR knockout pooled library from the Feng Zhang lab (Addgene catalog number 1000000049) at a MOI of 0.3 to limit cotransduction. The transduced cells were selected with puromycin (1 μg/mL) for 7 days to generate knockout cell pools. Input genomic DNA was extracted from 30 million cells to ensure the coverage of the library. About 120 million cells were treated with Dox (1 μg/mL) for 24 h to trigger KSHV lytic replication, followed by FACS analysis. Genomic DNA was extracted from the sorted cells with top 5% EGFP intensity. The sgRNA sequences were amplified using PrimeSTAR GXL DNA polymerase (TaKaRa) to prepare the sequencing library, and next-generation sequencing was carried out by Annoroad Gene Technology (Beijing, China). The MAGeCK algorithm was used to identify statistically significant hits.

### CUT&Tag analysis.

The CUT&Tag experiment was performed by using a hyperactive *in situ* ChIP Library Prep kit (pG-Tn5; Vazyme, Nanjing, China) according to the manufacturer’s guidelines.

In brief, SLK.iBAC-GFP cells were treated with Dox (1 μg/mL) for 24 h to induce lytic reactivation. About 10,000 SLK.iBAC cells were collected and bound to concanavalin A-coated magnetic beads, followed by permeabilization with digitonin. Then, the cells were incubated with SMCHD1 antibody or rabbit IgG (1 μg) for 2 h at room temperature and subsequently with the second antibody for 1 h. A hyperactive protein pG-Tn5 transposase fusion protein was added and activated to generate chromatin fragments. DNA was purified by phenol-chloroform extraction, and Illumina sequencing libraries were constructed for next-generation sequencing.

For analysis, sequencing reads were filtered by using Trim Galore, and clean reads were mapped to the human genome (hg19) using Bowtie2 with default parameters (50); unmapped reads were then mapped to the KSHV genome (GenBank accession number NC_009333.1) using Bowtie2 with the following parameters: –end-to-end –very-sensitive –no-mixed –no-discordant –phred33 -I 10 -X 700. The peaks on the KSHV genome were called using MACS2 software (51).

### ChIP assay.

SLK.iBAC-GFP cells were treated with Dox (1 μg/mL) for 24 h. BCBL-1-Tet-RTA cells were treated with Dox (1 μg/mL) and sodium butyrate (0.2 mM) for 24 h.

ChIP assays were performed as previously described (52). About 1 × 10^7^ SLK.iBAC or BCBL-1-Tet-RTA cells were used for the experiment. The cells were cross-linked with 1% formaldehyde for 20 min at room temperature, and glycine was added to a final concentration of 0.125 M to quench the reaction. The fixed cells were resuspended in cell lysis buffer (10 mM Tris-Cl [pH 8.0], 10 mM NaCl, 0.5% NP-40) supplemented with protease inhibitors and incubated on ice for 20 min. After centrifugation, the pellets were collected and resuspended in nuclear lysis buffer (50 mM Tris-Cl [pH 8.0], 10 mM EDTA,1% SDS) supplemented with protease inhibitors. Sonication shearing was carried out according to the manufacturer’s guidelines (Covaris S220). After centrifugation, the sonicated chromatin was diluted with dilution buffer (50 mM Tris-Cl [pH 8.0], 10 mM EDTA, 1% Triton X-100, 0.1% Na-deoxycholate) supplemented with protease inhibitors. Then, the samples were incubated overnight with 1 μg of the indicated antibody or IgG at 4°C. After that, 10 μL preconjugated magnetic protein A/G beads (Bio-Linkedin, Wuhan, China) was added to recover the antibody-antigen complex. The beads were washed sequentially with washing buffer I (20 mM Tris-HCl [pH 8.0], 2 mM EDTA [pH 8.0], 150 mM NaCl, 1% Triton X-100, 0.1% SDS, 1× proteinase inhibitor cocktail) 3 times, washing buffer II (20 mM Tris-HCl [pH 8.0], 2 mM EDTA [pH 8.0], 500 mM NaCl, 1% Triton X-100, 0.1% SDS, 1× proteinase inhibitor cocktail) 3 times, and washing buffer III (10 mM Tris-HCl [pH 8.0], 1 mM EDTA [pH 8.0], 250 mM LiCl, 1% NP-40, 1% Na-deoxycholate, 1× proteinase inhibitor cocktail) and TE buffer for 1 time, respectively. The binding DNA was eluted with 100 μL of elution buffer (10 mM Tris-Cl [pH 8.0], 1 mM EDTA, 1% SDS) and were incubated overnight at 65°C. The samples were then digested with protease K and RNase A, and DNA was purified by phenol-chloroform extraction.

The primers for qPCR analysis are provided in Table S2. A pair of primers amplifying the coding region of KSHV ORF20 was designed as a negative control.

### Quantification of KSHV titer.

SLK.iBAC-GFP cells were induced with Dox (1 μg/mL) and sodium butyrate (0.1 mM) to trigger lytic reactivation. The cell culture supernatants were collected at the indicated times postinduction and were used to infect HEK293T cells with appropriate dilution. After 24 h, the infected 293T cells were collected and subjected to flow cytometry analysis. Flow cytometry data were analyzed with FlowJo 10.0, and the KSHV titer was calculated based on GFP-positive cell percentage and is presented in IU per milliliter.

### KSHV *de novo* infection.

Approximately 4 × 10^4^ HUVEC were infected with KSHV in the presence of polybrene (8 μg/mL). The infected cells were washed twice with phosphate-buffered saline 6 h postinfection and were maintained in DMEM until harvesting. The KSHV-infected HUVEC were collected at the indicated time points, and total RNA and genomic DNA were extracted to analyze viral gene expression and viral genomic DNA.

### Quantification of viral genome copy number.

SLK.iBAC-GFP cells were treated with Dox (1 μg/mL) for 48 h; BCBL-1-Tet-RTA cells were treated with Dox (1 μg/mL) and sodium butyrate (0.2 mM) for 48 h; P3HR-1 cells were induced with PMA (25 ng/mL, MedChemExpress) and sodium butyrate (0.3 mM) for 48 h.

Genomic DNA from 3 × 10^5^ P3HR-1, SLK.iBAC-GFP, or BCBL-1-Tet RTA cells was extracted by phenol-chloroform extraction and was used for the quantification of intracellular viral DNA. Cell culture supernatant (500 μL) was collected and was treated with 7.5 U of DNase I (Solarbio, Beijing, China) for 1 h at 37°C to digest nonencapsidated viral genomes. Then, 30 μL of proteinase K (20 mg/mL; Solarbio, Beijing, China) and 50 μL of 20% SDS was added into the reaction mixture. After 1 h of incubation at 65°C, DNA was isolated by phenol-chloroform extraction and the purified DNA was resolved in 100 μL Tris-EDTA buffer. EBV genomic DNA was quantified by qPCR targeting the BZLF1 gene. Serial dilutions of a pEF-FLAG-BZLF1 plasmid were used to set up a stand curve for the qPCR analysis. KSHV genomic DNA was quantified by qPCR targeting the ORF50 gene. Serial dilutions of a pEGFP-RTA plasmid were used to set up a standard curve for the qPCR analysis.

The primers used for the quantification of viral genome copy number are provided in Table S2.

### Stable cell line generation.

Short hairpin RNA (shRNA) targeting SMCHD1 was kindly provided Chengyu Liang (Wistar Institute, Pennsylvania). sgRNA targeting SMCHD1 was constructed into Lenti-CRISPR v2 (Addgene). The following gRNA sequences were used in the study: sgSMCHD1 #1, 5′-GACGGCGGCGGGCCTGGTG-3′; sgSMCHD1 #2, 5′-ATCCTCCTCAGTCCCCACAG-3′.

Lentiviruses were produced in 293T cell as previously described (45, 53). SLK.iBAC-GFP, SLK.iBAC-ORF52-EGFP, BCBL-1, HUVEC, HFF, and P3HR-1 cells were infected with lentiviruses containing shRNA or sgRNA targeting SMCHD1. After 48 h, the transduced cells were selected with puromycin (1 μg/mL) for 2 days. SLK.iBAC-GFP SMCHD1 knockdown stable cells were stably reconstituted with vector, SMCHD1, or the mutants through lentiviral transduction. The reconstituted cells were selected with G418 (500 μg/mL) for 3 days. All stable cells were maintained in culture medium containing hygromycin B, blasticidin, puromycin, or G418.

### Biotin-labeled ORI-Lyt pulldown.

SMCHD1 and the mutants were transfected and transiently expressed in HEK293T cells, and whole-cell lysates were prepared 48 h posttransfection. The lysates or SMCHD1 hinge domain (aa 1682 to 1898) purified from Escherichia coli was incubated with biotinylated KSHV ORI-Lyt, HSV-1 ORI-Lyt, HCMV ORI-Lyt, MHV68 ORI-Lyt, or biotin-EGFP for 45 min at room temperature. Then, 10 μL of streptavidin beads (Thermo Fisher) was added, and the samples were rotated for 4 h at 4°C. The beads were washed with lysis buffer (50 mM Tris-Cl [pH 7.5], 150 mM NaCl, 1% Triton X-100, 1× proteinase inhibitors) three times, and the pulldown proteins were recovered by boiling with SDS sample buffer for 5 min at 95°C. Input and precipitated proteins were resolved by SDS-PAGE and analyzed by immunoblotting.

### Protein purification.

SMCHD1 hinge domain (aa 1682 to 1898) was expressed in Escherichia coli BL21-DE3 cells by induction with 0.5 mM isopropyl-β-d-thiogalactopyranoside at 25°C overnight. Cells were collected and sonicated in lysis buffer (50 mM Tris-Cl [pH 8.0], 150 mM NaCl, 10% glycerol, 10 mM imidazole, 1% Triton X-100, 0.2 mM phenylmethylsulfonyl fluoride). The supernatant was collected and incubated with Ni-nitrilotriacetic acid beads. After washing with buffer (50 mM Tris-Cl [pH 8.0], 150 mM NaCl, 10% glycerol, 20 mM imidazole) three times, bound protein was eluted with elution buffer (20 mM Tris-Cl [pH 7.4], 150 mM NaCl, 400 mM imidazole). The protein was used for pulldown assays.

### D4Z4 array methylation analysis by bisulfite conversion sequencing.

The assessment of D4Z4 DNA methylation was performed with an EpiArt DNA methylation bisulfite kit (Vazyme, Nanjing, China) according to the manufacturer’s guidelines.

In brief, 100 ng of genomic DNA from SLK.iBAC-GFP cells was converted using the kit. PCR analysis was carried out on the samples using the following primers: 5′-GTAGAGGGGATTTTTTAATTTGTTT-3′ and 5′-CAAACACCCCTTAACCCTAC-3′. Subsequently, the PCR product was digested, ligated into a vector, and transformed into DH5α competent cells. Eight colonies were picked for each sample, and plasmid DNA was extracted and sent for Sanger sequencing.

### Statistical analysis.

Data represent the means of at least three independent experiments, and error bars denote standard deviations (SD). A two-tailed Student's *t* test or analysis of variance (ANOVA) was used for statistical analysis. For all statistical analyses, asterisks are defined as follows: *, *P* < 0.05; **, *P* < 0.01; ***, *P* < 0.005.
