# Supplementary figures and images for: Genome-Wide CRISPR-Cas9 Screen Identifies SMCHD1 as a Restriction Factor for Herpesviruses
Source: mBio. 2023 Apr 3;14(2):e00549-23. doi: 10.1128/mbio.00549-23 (PMC10128004; doi:10.1128/mbio.00549-23)

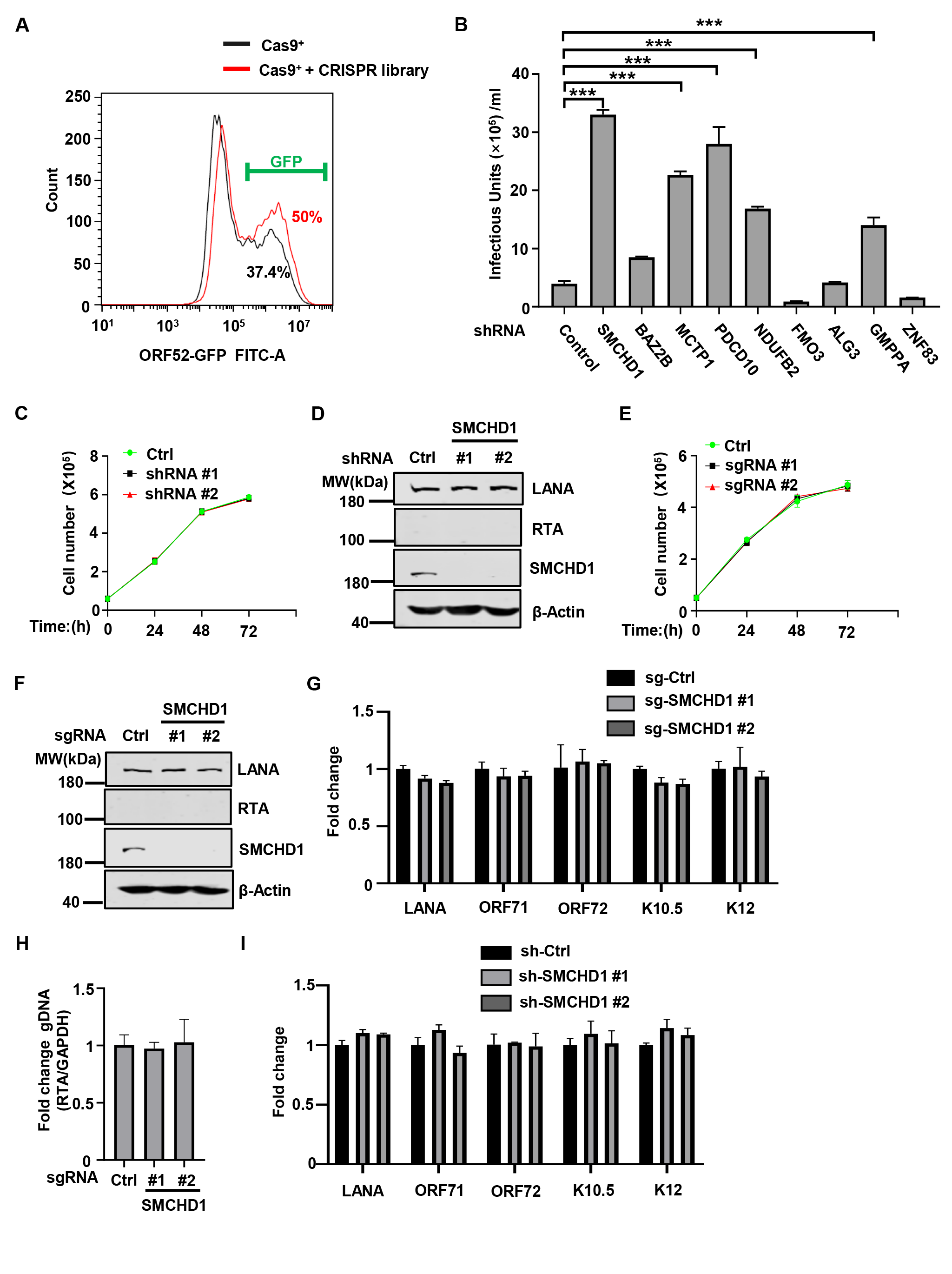

Supplement: FIG S1 [file mbio.00549-23-s0001.tif]

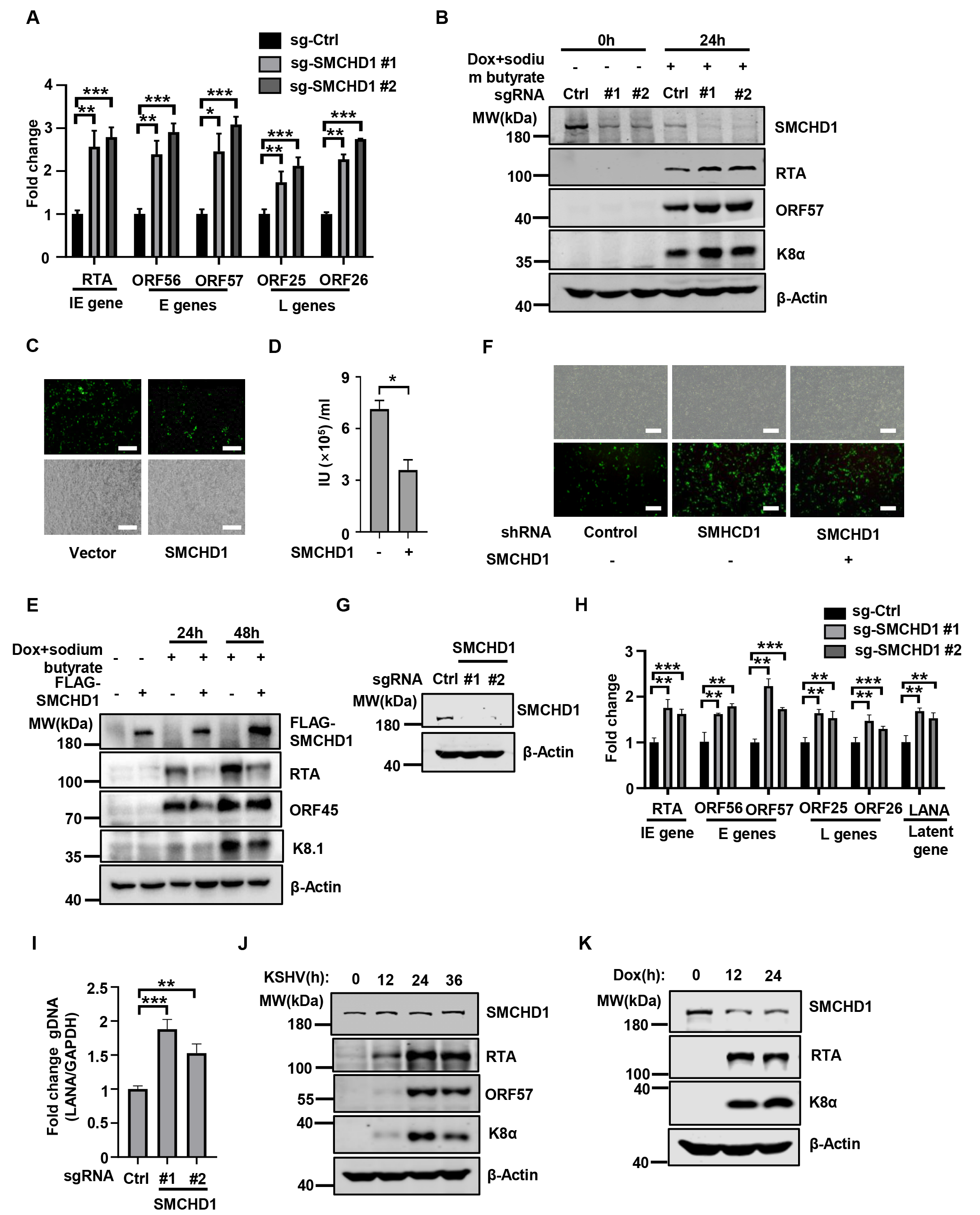

Supplement: FIG S2 [file mbio.00549-23-s0002.tif]

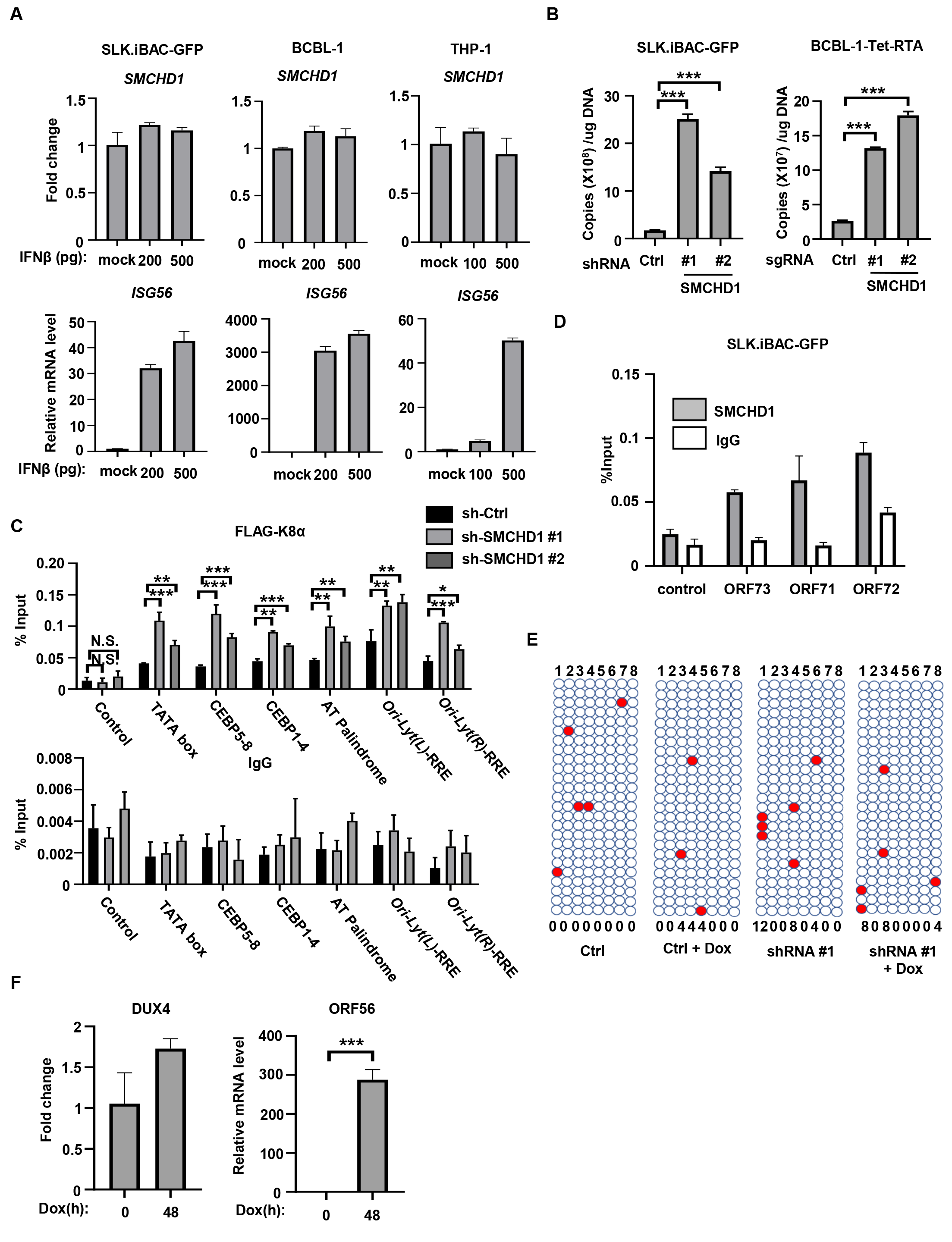

Supplement: FIG S3 [file mbio.00549-23-s0003.tif]

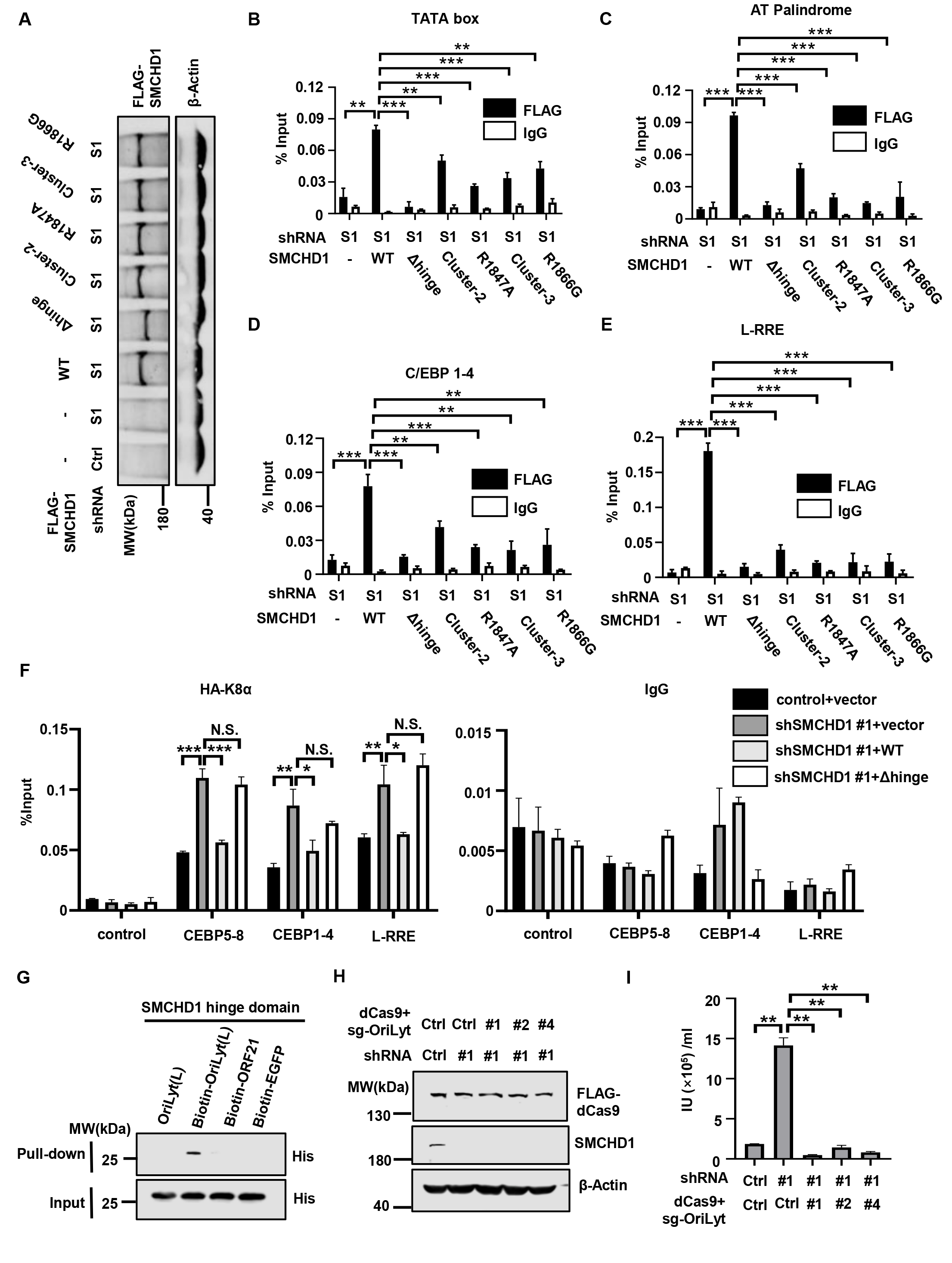

Supplement: FIG S4 [file mbio.00549-23-s0004.tif]

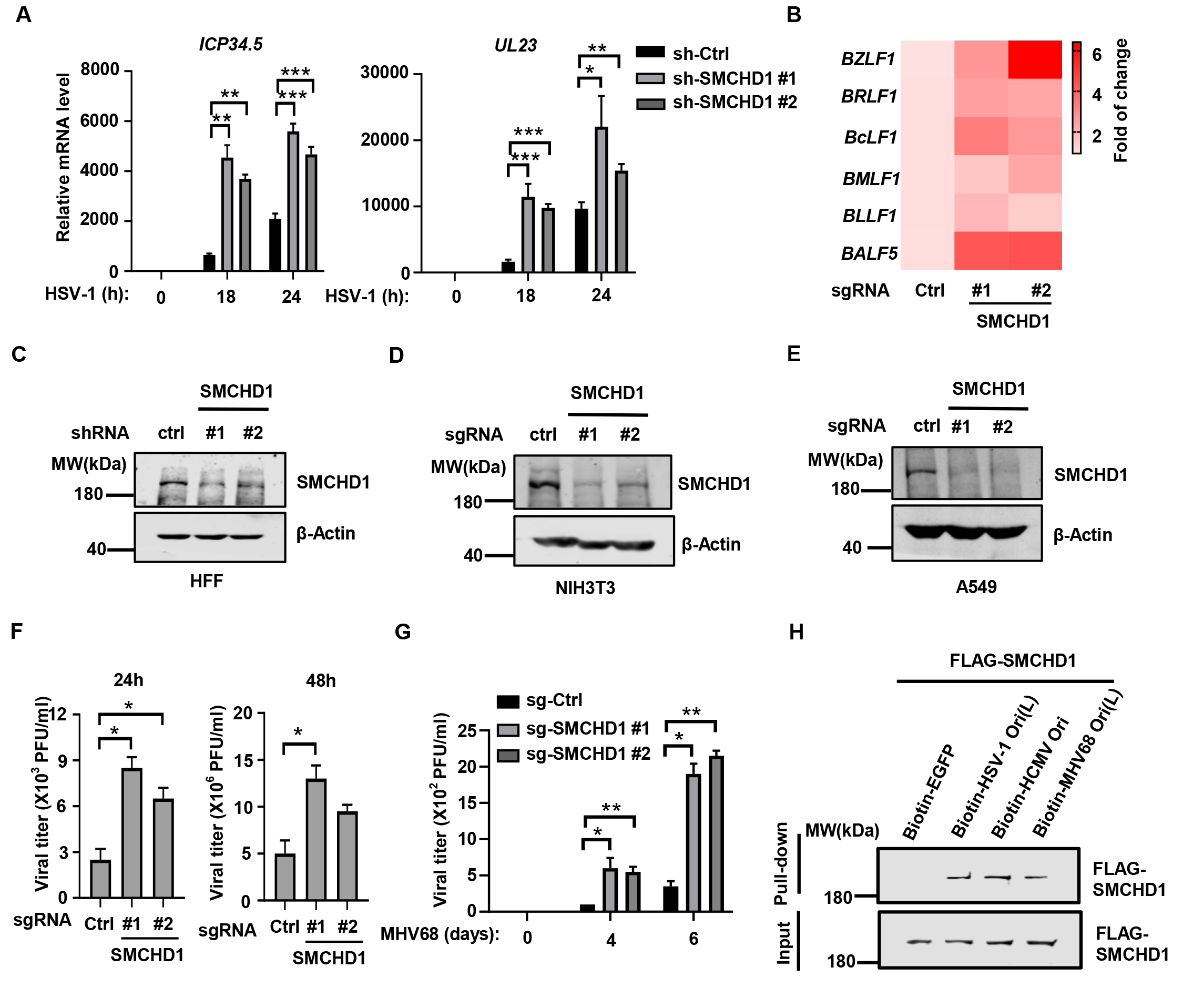

Supplement: FIG S5 [file mbio.00549-23-s0005.tif]
