# Supplementary material for: Genome-Wide CRISPR-Cas9 Screen Identifies SMCHD1 as a Restriction Factor for Herpesviruses
Source: mBio. 2023 Apr 3;14(2):e00549-23. doi: 10.1128/mbio.00549-23 (PMC10128004; doi:10.1128/mbio.00549-23)
Supplement: TABLE S1 [file mbio.00549-23-s0006.pdf]

id	num	neg|score	neg|p-val	neg|fdr	neg|rank	neg|	pos|score	pos|p-value	pos|fdr	pos|rank
SMCHD1	6	0.9904	0.9903	0.999	21613	0	3.62E-08	6.81E-07	0.0149	1
BAZ2B	6	0.3825	0.5791	0.997	12512	3	4.68E-07	3.86E-06	0.0421	2
MCTP1	6	0.1673	0.3381	0.997	7324	2	2.51E-05	0.00012912	0.6327	3
PDCD10	6	0.9392	0.9388	0.998	20513	0	2.57E-05	0.00013139	0.6327	4
NDUFB2	6	0.9973	0.9973	1	21760	0	2.81E-05	0.00014501	0.6327	5
FMO3	6	0.9989	0.9989	1	21796	0	4.69E-05	0.00023306	0.6505	6
hsa-mir-509	4	0.2174	0.3968	0.997	8565	2	5.02E-05	0.00024804	0.6505	7
ALG13	6	0.9999	0.9999	1	21815	0	6.56E-05	0.00031611	0.6505	8
GMPPA	6	0.1501	0.3177	0.997	6881	2	6.67E-05	0.00032065	0.6505	9
ZNF83	6	0.6796	0.7848	0.997	17069	1	6.71E-05	0.00032201	0.6505	10
hsa-mir-52	4	0.9633	0.963	0.999	21030	0	7.90E-05	0.00037648	0.6505	11
C10orf2	6	0.9999	0.9999	1	21814	0	8.14E-05	0.00038737	0.6505	12
STK17A	6	0.7819	0.8379	0.997	18244	1	8.38E-05	0.0003969	0.6505	13
MYL12B	6	0.9932	0.9931	0.999	21684	0	9.30E-05	0.00044229	0.6505	14
TRIM68	6	0.6952	0.7965	0.997	17329	1	9.37E-05	0.00044728	0.6505	15
ETFB	6	0.9262	0.9265	0.997	20242	1	0.000106	0.00050946	0.6946	16
RBMS3	6	0.2605	0.4461	0.997	9565	2	0.000126	0.00060386	0.7749	17
RASA4B	3	0.9999	0.9999	1	21813	0	0.000139	0.00067376	0.8166	18
GNG5	6	0.3658	0.5613	0.997	12142	1	0.000158	0.00076498	0.858	19
CNOT2	6	0.4853	0.65	0.997	14092	1	0.000172	0.00083125	0.858	20
HAS1	6	0.8112	0.8517	0.997	18552	1	0.000176	0.00084986	0.858	21
SLC30A9	6	0.7392	0.8199	0.997	17855	1	0.000178	0.00086529	0.858	22
C2orf72	6	0.2151	0.3941	0.997	8508	2	0.000191	0.0009379	0.8675	23
TCP11	6	0.9992	0.9992	1	21799	0	0.000199	0.00098284	0.8675	24
SPDYE1	4	0.9998	0.9998	1	21812	0	0.000201	0.00099418	0.8675	25
CRAMP1L	6	0.9998	0.9998	1	21811	0	0.000224	0.0011113	0.9081	26
PLXNC1	6	0.6352	0.7522	0.997	16381	1	0.000226	0.001124	0.9081	27
GDI2	6	0.9875	0.9874	0.999	21554	0	0.000266	0.0013241	0.9316	28
ABCF1	6	0.9339	0.9336	0.997	20418	1	0.000276	0.0013713	0.9316	29
hsa-mir-60	4	0.309	0.4998	0.997	10755	2	0.000283	0.0014036	0.9316	30
SHISA4	6	0.2554	0.4404	0.997	9447	2	0.000283	0.001404	0.9316	31
TMLHE	6	0.9803	0.98	0.999	21388	0	0.00029	0.0014335	0.9316	32
TAS1R1	6	0.9257	0.926	0.997	20234	1	0.000298	0.0014689	0.9316	33
PLGLB2	4	0.9997	0.9997	1	21810	0	0.000322	0.0015774	0.9316	34
EFHC2	6	0.8659	0.8819	0.997	19213	1	0.000325	0.001586	0.9316	35
JAKMIP2	6	0.2701	0.4569	0.997	9847	3	0.000326	0.0015883	0.9316	36
TRIM3	6	0.9872	0.9871	0.999	21544	0	0.000364	0.001778	0.9316	37
EED	6	0.9197	0.9208	0.997	20108	1	0.000377	0.0018347	0.9316	38
ZNF35	6	0.9816	0.9814	0.999	21420	0	0.000383	0.001857	0.9316	39
NKAPL	6	0.9996	0.9996	1	21809	0	0.000389	0.0018842	0.9316	40
NUP98	6	0.9986	0.9986	1	21788	0	0.000398	0.0019364	0.9316	41
SCAMP5	6	0.4869	0.651	0.997	14110	2	0.000422	0.002053	0.9316	42
ZC3H13	6	0.9981	0.9981	1	21776	0	0.000426	0.0020744	0.9316	43
ZNF544	6	0.1272	0.2845	0.997	6175	3	0.000427	0.0020762	0.9316	44
CRISP2	6	0.5732	0.7088	0.997	15394	2	0.000436	0.0021229	0.9316	45
HBEGF	6	0.9854	0.9852	0.999	21503	0	0.000458	0.0022196	0.9316	46
FAM220A	6	0.9839	0.9837	0.999	21475	0	0.00046	0.0022319	0.9316	47

PLCH1	6	0.9995	0.9995	1	21808	0	0.000466	0.0022655	0.9316	48
KRT73	6	0.8882	0.8965	0.997	19543	1	0.000477	0.0023149	0.9316	49
FGF20	6	0.086	0.2089	0.997	4500	2	0.000477	0.0023176	0.9316	50
SPATA31E1	6	0.9995	0.9995	1	21807	0	0.00048	0.0023326	0.9316	51
OTUD7B	6	0.8593	0.8779	0.997	19124	1	0.000492	0.0023912	0.9316	52
SLC4A10	6	0.8318	0.8623	0.997	18776	1	0.000498	0.0024141	0.9316	53
STOML1	6	0.9603	0.96	0.999	20957	0	0.000498	0.0024166	0.9316	54
GNA13	6	0.8421	0.868	0.997	18913	1	0.000517	0.0025062	0.9316	55
TRIM69	6	0.9995	0.9995	1	21806	0	0.000527	0.0025532	0.9316	56
PAK6	6	0.0606	0.1594	0.996	3489	2	0.000527	0.0025582	0.9316	57
LILRA2	6	0.1558	0.3245	0.997	7011	1	0.000534	0.0025822	0.9316	58
EQTN	6	0.0116	0.0419	0.972	914	3	0.000547	0.0026358	0.9316	59
ZNF446	6	0.2088	0.3869	0.997	8355	1	0.000555	0.0026785	0.9316	60
CBX4	6	0.9974	0.9974	1	21763	0	0.000579	0.0028024	0.9316	61
COG8	6	0.0158	0.055	0.972	1195	3	0.0006	0.0028954	0.9316	62
KCNC3	6	0.7125	0.8097	0.997	17619	1	0.000603	0.0029009	0.9316	64
SFXN3	6	0.9984	0.9984	1	21783	0	0.000603	0.0029009	0.9316	63
MMP16	6	0.2733	0.4604	0.997	9937	3	0.000606	0.0029172	0.9316	65
OPN1MW	4	0.9931	0.993	0.999	21682	0	0.000626	0.0030089	0.9316	66
DGAT2	6	0.9174	0.9188	0.997	20062	1	0.000635	0.0030556	0.9316	67
HOXC6	6	0.1394	0.3046	0.997	6592	3	0.000637	0.0030638	0.9316	68
CA1	6	0.5427	0.6879	0.997	14920	1	0.000638	0.0030652	0.9316	69
PHOX2B	6	0.6905	0.7929	0.997	17247	1	0.000653	0.0031396	0.9316	70
TMEM242	6	0.9993	0.9993	1	21805	0	0.000661	0.0031723	0.9316	71
PRRC1	6	0.9993	0.9993	1	21804	0	0.000673	0.003229	0.9316	72
hsa-mir-54[illegible]	4	0.9993	0.9993	1	21803	0	0.000676	0.0032422	0.9316	73
OR3A2	6	0.0051	0.0204	0.972	436	2	0.000678	0.0032481	0.9316	74
MRPL52	6	0.8967	0.9026	0.997	19682	1	0.000692	0.003318	0.9316	75
UNC119B	6	0.9993	0.9993	1	21802	0	0.000715	0.0034169	0.9316	76
HOMER3	6	0.0596	0.1574	0.994	3450	2	0.000728	0.0034786	0.9316	77
SULT4A1	6	0.9887	0.9886	0.999	21574	0	0.000738	0.0035204	0.9316	78
IL1RAPL2	6	0.8264	0.8595	0.997	18714	1	0.000769	0.0036602	0.9316	79
FAM178A	6	0.8695	0.8841	0.997	19259	1	0.000771	0.0036665	0.9316	80
BIRC2	6	0.0021	0.0097	0.934	225	4	0.000778	0.0036987	0.9316	81
DGUOK	6	0.3719	0.5678	0.997	12272	1	0.000787	0.0037378	0.9316	83
CLDN20	6	0.757	0.8271	0.997	18017	1	0.000787	0.0037378	0.9316	82
TNR	6	0.9966	0.9966	1	21745	0	0.00079	0.0037487	0.9316	84
DECR1	6	0.9992	0.9992	1	21801	0	0.000791	0.0037496	0.9316	85
hsa-mir-80[illegible]	4	0.6935	0.7952	0.997	17297	1	0.000795	0.0037695	0.9316	86
PYGL	6	0.9992	0.9992	1	21800	0	0.00082	0.0038817	0.9316	87
LCN9	6	0.8679	0.8832	0.997	19237	1	0.000822	0.0038921	0.9316	88
NPAS1	6	0.8968	0.9026	0.997	19683	1	0.000828	0.0039234	0.9316	89
MAFF	6	0.327	0.5196	0.997	11171	3	0.000828	0.003927	0.9316	90
ATXN7L3B	6	0.0677	0.1735	0.997	3762	2	0.000842	0.0039983	0.9316	91
KALRN	6	0.1075	0.2492	0.997	5378	2	0.000846	0.0040183	0.9316	92
MLF1	6	0.6959	0.797	0.997	17341	2	0.000849	0.004031	0.9316	93
hsa-mir-59[illegible]	3	0.8984	0.9038	0.997	19710	0	0.000854	0.0040523	0.9316	94
TAS2R39	6	0.7565	0.8269	0.997	18009	1	0.000856	0.0040605	0.9316	95

B4GALT6	6	0.2117	0.3903	0.997	8429	3	0.000879	0.0041721	0.9316	96
CNTN4	6	0.0429	0.123	0.982	2722	4	0.000882	0.0041853	0.9316	97
CHGB	6	0.2603	0.4459	0.997	9562	1	0.000895	0.0042452	0.9316	98
hsa-mir-43:	4	0.9991	0.9991	1	21798	0	0.000904	0.0042742	0.9316	99
SMCR8	6	0.3863	0.5832	0.997	12613	2	0.000929	0.0043895	0.9316	100
GJA3	6	0.9669	0.9666	0.999	21097	0	0.000944	0.0044621	0.9316	101
GPSM1	6	0.4561	0.6306	0.997	13659	1	0.000951	0.0044925	0.9316	102
LRRC3C	6	0.6074	0.7327	0.997	15949	2	0.000956	0.004513	0.9316	103
HMGCS1	6	0.999	0.999	1	21797	0	0.000961	0.0045307	0.9316	104
ORMDL1	6	0.0262	0.0855	0.976	1908	1	0.000968	0.0045683	0.9316	105
C3	6	0.267	0.4534	0.997	9778	2	0.000975	0.004591	0.9316	106
NCDN	6	0.5191	0.6721	0.997	14558	2	0.000975	0.004591	0.9316	107
MOGS	6	0.1989	0.3753	0.997	8091	3	0.000979	0.0046119	0.9316	108
PARG	6	0.7203	0.8126	0.997	17689	1	0.000999	0.0046941	0.9388	109
SRGAP2B	2	0.3522	0.5469	0.997	11815	1	0.001013	0.0047612	0.9388	110
hsa-mir-68	4	0.9222	0.9229	0.997	20159	0	0.001023	0.0048075	0.9388	111
MTFP1	6	0.6059	0.7317	0.997	15914	2	0.001029	0.0048366	0.9388	112
SMAD3	6	0.898	0.9035	0.997	19702	1	0.001047	0.0049183	0.9388	113
HGFAC	6	0.9774	0.9771	0.999	21317	0	0.001066	0.0050022	0.9388	114
PRDM5	6	0.4706	0.6404	0.997	13875	1	0.001072	0.0050354	0.9388	115
UNC50	6	0.764	0.8301	0.997	18083	1	0.001079	0.0050694	0.9388	116
MAN1C1	6	0.4256	0.6099	0.997	13202	2	0.001082	0.0050785	0.9388	117
SSB	6	0.0883	0.2133	0.997	4591	1	0.001118	0.005246	0.9388	118
ZNF578	5	0.9989	0.9989	1	21795	0	0.001126	0.0052823	0.9388	119
MYH1	6	0.9989	0.9989	1	21794	0	0.001127	0.0052854	0.9388	120
ZNF407	6	0.2112	0.3896	0.997	8406	3	0.001127	0.0052864	0.9388	121
UTP20	6	0.6809	0.7857	0.997	17081	2	0.00113	0.0052982	0.9388	122
hsa-mir-39:	4	0.879	0.8902	0.997	19407	0	0.001135	0.0053249	0.9388	123
AK2	6	0.9989	0.9989	1	21793	0	0.001148	0.0053798	0.9388	124
PTK6	6	0.9341	0.9338	0.997	20420	1	0.00118	0.005526	0.9388	125
LOC10012!	6	0.9988	0.9988	1	21792	0	0.001209	0.0056381	0.9388	126
CA7	6	0.9218	0.9226	0.997	20150	1	0.001214	0.005659	0.9388	127
NEDD4L	6	0.9949	0.9949	0.999	21721	0	0.001218	0.0056726	0.9388	128
DGKG	6	0.9596	0.9593	0.999	20943	0	0.00123	0.0057239	0.9388	129
ARL2BP	6	0.7226	0.8135	0.997	17711	1	0.00123	0.0057266	0.9388	130
ZFX	6	0.3049	0.4954	0.997	10661	2	0.001244	0.0057901	0.9388	131
EXOC6	6	0.1558	0.3245	0.997	7025	1	0.001246	0.0057947	0.9388	132
ZNF555	6	0.2134	0.3922	0.997	8467	2	0.00126	0.0058578	0.9388	133
KPTN	6	0.9665	0.9662	0.999	21092	0	0.001262	0.00587	0.9388	134
WTAP	5	0.9944	0.9943	0.999	21706	0	0.001276	0.0059254	0.9388	135
MYPN	6	0.5124	0.6677	0.997	14456	2	0.00128	0.0059494	0.9388	136
TM2D1	6	0.9208	0.9217	0.997	20128	1	0.001297	0.0060198	0.9388	137
BTAF1	6	0.9941	0.994	0.999	21697	0	0.00131	0.0060724	0.9388	138
HNRNPL	6	0.9943	0.9943	0.999	21705	0	0.00132	0.006126	0.9388	139
NonTargeti	1	0.9987	0.9987	1	21791	0	0.001327	0.0061514	0.9388	140
BAZ1A	6	0.0256	0.0838	0.975	1874	3	0.00133	0.0061655	0.9388	141
RALGDS	6	0.697	0.7979	0.997	17363	2	0.001336	0.0061977	0.9388	142
hsa-mir-36	4	0.5514	0.6938	0.997	15026	1	0.00134	0.006214	0.9388	143

C8orf44-SG	5	0.9987	0.9987	1	21790	0	0.001347	0.0062467	0.9388	144
TUBG1	5	0.9041	0.9081	0.997	19811	0	0.001354	0.0062767	0.9388	145
TMEM211	6	0.8417	0.8678	0.997	18907	1	0.001355	0.006283	0.9388	146
GK	6	0.7335	0.8176	0.997	17798	1	0.00138	0.0064056	0.9428	147
hsa-mir-93	4	0.9986	0.9986	1	21789	0	0.001385	0.0064178	0.9428	148
DCLRE1C	6	0.304	0.4943	0.997	10644	2	0.00139	0.0064396	0.9428	149
FBXO31	6	0.943	0.9428	0.998	20607	0	0.00141	0.0065231	0.9487	150
PDK4	6	0.5647	0.7029	0.997	15244	2	0.001431	0.0066089	0.9503	151
ROR1	6	0.6059	0.7317	0.997	15923	2	0.001449	0.0066938	0.9503	152
KIAA0368	6	0.6543	0.766	0.997	16663	1	0.001457	0.0067314	0.9503	153
ZNF514	6	0.0001	0.0006	0.811	14	3	0.001481	0.0068381	0.9503	154
VTI1B	6	0.5449	0.6895	0.997	14947	1	0.001492	0.0068835	0.9503	155
ENTPD4	6	0.2154	0.3944	0.997	8517	2	0.001505	0.0069416	0.9503	156
C2orf16	6	0.9985	0.9985	1	21787	0	0.001522	0.0070206	0.9503	157
hsa-mir-12	4	0.1353	0.2988	0.997	6474	1	0.001522	0.0070228	0.9503	158
CRYGS	6	0.9892	0.9891	0.999	21583	0	0.001529	0.0070537	0.9503	159
OR8K3	6	0.9985	0.9985	1	21786	0	0.00153	0.0070591	0.9503	160
KIAA1239	6	0.5243	0.6756	0.997	14631	2	0.001531	0.0070632	0.9503	161
MCM5	6	0.142	0.3079	0.997	6671	1	0.001541	0.0071109	0.9503	162
ZCCHC8	6	0.6137	0.737	0.997	16039	2	0.001567	0.0072271	0.9503	163
CNNM4	6	0.9984	0.9984	1	21785	0	0.001568	0.0072316	0.9503	164
PRSS27	6	0.9984	0.9984	1	21784	0	0.00157	0.0072398	0.9503	165
KRT5	6	0.2447	0.4283	0.997	9220	3	0.001606	0.0074159	0.9503	166
CACNA1H	6	0.3953	0.5898	0.997	12739	2	0.001606	0.0074159	0.9503	167
WDR5B	6	0.9984	0.9984	1	21782	0	0.001622	0.0074889	0.9503	168
LOC10012	6	0.6153	0.7382	0.997	16064	2	0.001641	0.007572	0.9503	169
C7orf60	6	0.2286	0.4098	0.997	8857	1	0.001647	0.007606	0.9503	170
ARMC12	6	0.8609	0.8789	0.997	19147	1	0.001649	0.0076115	0.9503	171
hsa-mir-13	4	0.9805	0.9802	0.999	21393	0	0.001656	0.0076464	0.9503	172
TJP1	6	0.1723	0.344	0.997	7458	3	0.001662	0.0076746	0.9503	173
CRIM1	6	0.6905	0.7929	0.997	17238	1	0.00168	0.0077386	0.9503	174
C1orf101	6	0.6764	0.7823	0.997	17024	1	0.001707	0.0078625	0.9503	175
TMEM26	6	0.9295	0.9294	0.997	20325	1	0.001708	0.0078684	0.9503	176
E2F3	6	0.9983	0.9983	1	21781	0	0.001713	0.0078902	0.9503	177
RFTN2	6	0.0385	0.1136	0.979	2516	4	0.001735	0.0079905	0.9503	178
EDDM3A	6	0.9103	0.9129	0.997	19924	1	0.001743	0.0080358	0.9503	179
PANX1	6	0.8684	0.8835	0.997	19246	1	0.00175	0.0080653	0.9503	180
IRF8	6	0.136	0.2999	0.997	6506	2	0.001752	0.0080749	0.9503	181
NDUFV2	6	0.7443	0.8219	0.997	17895	1	0.001757	0.0081012	0.9503	182
PSIP1	6	0.9958	0.9958	0.999	21733	0	0.001764	0.0081262	0.9503	183
ZBTB17	6	0.9675	0.9673	0.999	21115	0	0.001768	0.0081516	0.9503	184
CDKN2D	6	0.466	0.6373	0.997	13818	2	0.001781	0.0082047	0.9503	185
UBIAD1	6	0.9982	0.9982	1	21780	0	0.001781	0.0082065	0.9503	186
PWWP2B	6	0.5977	0.7258	0.997	15772	2	0.001785	0.0082242	0.9503	187
hsa-mir-12	4	0.879	0.8902	0.997	19410	0	0.00179	0.0082433	0.9503	188
AGPAT4	6	0.9982	0.9982	1	21779	0	0.001791	0.0082469	0.9503	189
hsa-mir-60	4	0.8424	0.8682	0.997	18915	0	0.001824	0.0084071	0.9503	190
N4BP2	6	0.9801	0.9798	0.999	21381	0	0.001832	0.0084416	0.9503	191

NAV3	6	0.9982	0.9982	1	21778	0	0.001847	0.0085133	0.9503	192
ZNF428	6	0.2739	0.4611	0.997	9960	2	0.001857	0.0085528	0.9503	193
DNAI2	6	0.4522	0.6279	0.997	13597	1	0.001857	0.0085528	0.9503	194
NUCKS1	6	0.8498	0.8724	0.997	19001	1	0.001857	0.0085528	0.9503	195
HSPA9	6	0.941	0.9406	0.998	20557	0	0.001861	0.0085732	0.9503	196
ZBTB20	6	0.3516	0.5463	0.997	11801	1	0.001863	0.0085814	0.9503	197
PTGR2	6	0.9981	0.9981	1	21777	0	0.001893	0.0087103	0.9566	198
SHC3	6	0.8942	0.9007	0.997	19640	1	0.001905	0.0087697	0.9566	199
ASPA	6	0.0158	0.055	0.972	1197	3	0.001906	0.0087716	0.9566	200
