# Supplementary material for: Genome-Wide CRISPR-Cas9 Screen Identifies SMCHD1 as a Restriction Factor for Herpesviruses
Source: mBio. 2023 Apr 3;14(2):e00549-23. doi: 10.1128/mbio.00549-23 (PMC10128004; doi:10.1128/mbio.00549-23)
Supplement: TABLE S2 [file mbio.00549-23-s0007.pdf]

Table S2 Primers used in this study

Real-time PCR primers		
KSHV RTA	CGCAATGCGTTACGTTGTTG	GCCCGGACTGTTGAATCG
KSHV ORF56	CACAGATTCCCGTCAATACAAA	GTATCTTCAGTAGGCGGCAGAG
KSHV ORF57	CATCCTAGAGGACTCTGT	TTGCTCGTCTTCCAGTGT
KSHV ORF25	ACAGTTTATGGCACGCATAGTG	GGTTCTCTGAATCTCGTCGTGT
KSHV ORF26	GCTCGAATCCAACGGATTTG	AATAGCGTGCCCCAGTTGC
KSHV LANA	CCTGGAAGTCCCACAGTGTT	AGACACAGGATGGGATGGAG
KSHV ORF72	TCTTGAAATTGAGCCGCGCT	CAACGCGACCACGTTAGGTT
KSHV ORF71	TAATTGGAGCTCTTAGAGC	ACGCGGGTCTAAGTGAAGCA
KSHV K10.5	TACACAGTGGGTCATCACTA	TGACCGGCACATCGCAAAAG
KSHV K12	TAACGGTGTTTGTGGCAGTT	CTCGTGTCCTGAATGCTACG
HSV-1 ICP0	GTCGCCTTACGTGAACAAGAC	GTCGCCATGTTTCCCGTCTG
HSV-1 UL23	GGAGGACAGACACATCGACC	TATTGGCAAGCAGCCCGTAA
HSV-1 ICP34.5	GTCCCAGGTAACCTCCACG	GACGCGGACTCGGGAAC
EBV BZLF1	AGGCCAGCTAACTGCCTATC	TGATTCTGGGTTATGTCGGA
EBV BRLF1	ACACTCCCGGCTGTAAATTC	TGGCTTGGAAGACTTTCTGA
EBV BALF5	AACCTTTGACTCGACCATCG	ACCTGCTCTTCGATGCACTT
EBV BcLF1	CATCCATGTTCATTGGGACC	CATTAGTCATACCTGCCAGG
EBV BLLF1	GTCAGTACACCATCCAGAGCC	TTGGTAGACAGCCTTCGTATG
EBV BMRF1	GCCGTTGAGGCCCACGTTGT	TGGGAATGGCAGGCGAGGGT
HCMV UL122	AAGATGTCCTGGCAGAAC	GAGGAGTGTTAGTAACCG
HCMV UL123	AAGATGTCCTGGCAGAAC	CTGCAAACATCCTCCCAT
HCMV UL78	TAGCCTGGTCAACCTGCTG	CCCTTGGACAACATGGTG
HCMV UL55	TTGGAGCGCGCAGTAGTGAT	CAACACCCACAGTACCCGTT
HCMV UL32	CAGCTTGCAGCGCTCCTGAA	GCCGTCAACAAGCTCGTGTA
HCMV UL94	CTCGTGTATCTTATGCCAG	GGTAGGTAGATGACAGAT
HCMV-gDNA	AAACCCCGACACGTACCGT	CGTCCTTGACACGATGGAGT
MHV68 ORF50	AGAAACCCACAGCTCGCACTT	CAATATGCTGGACAGGCGTATC
MHV68 ORF9	TGCATGCAAGTTTGTCCAGTCT	CTTCCCCCAGTTACTCATTGTTTG
MHV68 ORF25	ACGTGCCAAGTGAATTCATG	TTCATGTGCATGGCCAACAT
Human β-actin	GTTGTCGACGACGAGCG	GCACAGAGCCTCGCCTT
Mouse β-actin	TCTACGAGGGCTATGCTCTCC	TCTTTGATGTCACGCACGATTTC
ChIP-qPCR primers		
TATA box	GGGACCGTGAGCGACTCGAA	GGGAGAGGGGCAGAGGAT
C/EBP 5-8	TCTGCCCCTCTCCCATTG	TAGCTCGAACGGGATTGGT
C/EBP 1-4	ATTGGACGCCCCAGCCGTCAAT	ATTGGTTCCCGCTGTGGGCCAAT
AT-Palindrome	ACCAATCAGCGATTAGAGT	GGGTATACCTACTGGAATA
Ori-Lyt(L)-RRE	CTGGCGCGCCCCAGAACAT	GGGCTGCTGTCCCTCGTTT
Ori-Lyt(R)-RRE	GGGGTCGGTCTCCCCTCTT	TGGCGCGGCCCCGGGAAAT
control	TCCAGCTCTAGGGAAAGCA	ACAGAGGCGGGAAGATTCT
